# 5-Alkylresorcinol Derivatives from the Bryozoan *Schizomavella mamillata*: Isolation, Synthesis, and Antioxidant Activity

**DOI:** 10.3390/md15110344

**Published:** 2017-11-02

**Authors:** María J. Ortega, Juan J. Pantoja, Carolina de los Reyes, Eva Zubía

**Affiliations:** Departamento de Química Orgánica, Facultad de Ciencias del Mar y Ambientales, 11510 Puerto Real (Cádiz), Spain; mariajesus.ortega@uca.es (M.J.O.); juanjose.pantoja@uca.es (J.J.P.); carolina.dereyes@uca.es (C.d.l.R.)

**Keywords:** alkylresorcinols, bryozoans, *Schizomavella*, 1,2-dihydrocyclobutabenzene, olefination, antioxidant

## Abstract

The chemical study of the bryozoan *Schizomavella mamillata* has led to the isolation of six new 5-alkylresorcinol derivatives, schizols A–F (**1**–**6**), whose structures were established by spectrocospic means. Schizol A (**1**) exhibits a (*E*)-6-phenylnon-5-enyl moiety linked to the C-5 of a resorcinol ring, while in schizol B (**2**) the substituent at C-5 contains an unusual 1,2-dihydrocyclobutabenzene moiety. Schizols C (**3**) and D (**4**) have been characterized as the 1-sulfate derivatives of **1** and **2**, respectively, and schizols E (**5**) and F (**6**) are the corresponding 1,3-disulfates. Schizol A (**1**) has been synthetized from 3,5-dimethoxybenzaldehyde through a sequence involving a Wittig reaction for the construction of the C-1′,C-2′ bond and a Julia–Kocienski olefination for the synthesis of the C-5′,C-6′ double bond. In the ABTS (2,2′-azinobis(3-ethylbenzothiazoline-6-sulphonic acid)) antioxidant assay, the natural compounds schizol A (**1**) and schizol B (**2**) showed higher radical scavenging activity than the Trolox standard.

## 1. Introduction

Bryozoans are sessile aquatic invertebrates, with most of the known species living in the marine environment [1]. These filter-feeding organisms typically form colonies that thrive on rocks near the coasts and coral reefs, but also on man-made structures such as piers and ships hulls. The phylum Bryozoa has been much less studied from a natural products perspective than other marine invertebrates such as sponges or coelenterates, and only about 220 new natural products have been isolated from bryozoans [2]. The compounds more often described have been alkaloids, mainly brominated alkaloids of diverse structural types that have been obtained from a few bryozoan species of the families Vesiculariidae and Flustridae [3,4,5,6,7,8]. However, the most renowned bryozoan-derived compounds are the bryostatins, a large series of macrocyclic lactones among which bryostatin-1 has reached clinical trials as an anticancer agent [9] and more recently as a treatment for Alzheimer’s disease [10]. Interestingly, while bryostatins were first isolated from the bryozoan *Bugula neritina* (family Bugulidae) [11], they were later shown to be produced by the symbiotic bacterium “*Candidatus* Endobugula sertula” [12].

As a part of our research on bioactive natural products from marine invertebrates of the southern coasts of Spain, we have studied samples of the bryozoan *Schizomavella mamillata* (Hincks, 1880), which belongs to the family Bitectiporidae. Previous chemical studies of this family of bryozoans have been limited to the species *Pentapora fascialis*, which was the source of pentaporins A–C [13]. These metabolites consist of two 5-alkylresorcinol-derived moieties linked through a disulfide bridge. The present study of *S. mamillata* has led to the isolation of six unprecedented 5-alkylresorcinol derivatives, schizols A–F (**1**–**6**). In addition, we describe synthetic routes for the obtention of schizol A (**1**) and its geometrical isomer **1′**. The isolated compounds (**1**–**6**) have also been tested in the ABTS (2,2′-azinobis(3-ethylbenzothiazoline-6-sulphonic acid)) radical-scavenging assay to assess their antioxidative properties.

## 2. Results and Discussion

### 2.1. Isolation and Structure Determination

**Figure 1:**
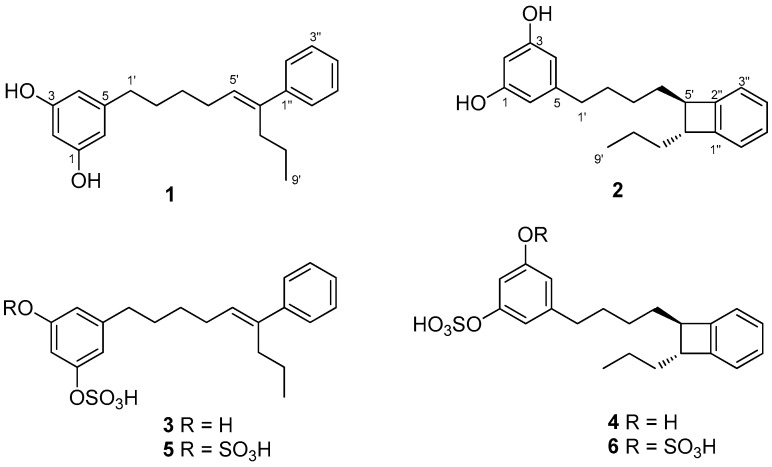
Chemical structures of schizols A–F (**1**–**6**).

Frozen specimens of *S. mamillata* were extracted with acetone/MeOH and, after evaporation of the solvent, the residue was extracted with Et_2_O and then with *n*-butanol. The Et_2_O extract and the *n*-butanol extract were separately subjected to column chromatography on silica gel. Further separation of fractions eluted with hexane/Et_2_O (3:7, *v*/*v*), Et_2_O, and CHCl_3_/MeOH (7:3, *v*/*v*) by using SPE-C18 cartridges and HPLC led to the isolation of compounds **1**–**6** (Figure 1).

**Figure 2:**
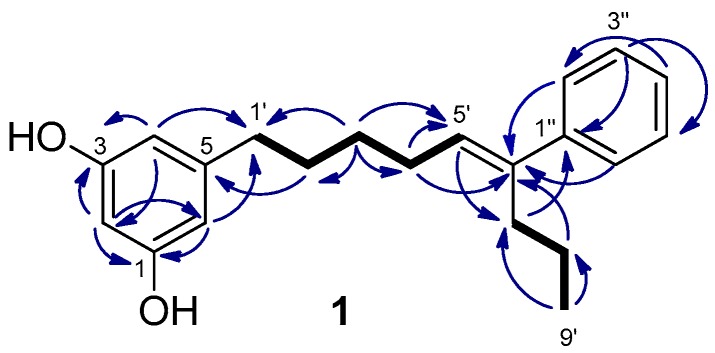
Key COSY (bold bond) and HMBC (→) correlations observed for schizol A (**1**).

Schizol A (**1**) was obtained as a colorless oil whose molecular formula C_21_H_26_O_2_ was established by HRESIMS(−). The IR absorption at 3365 cm^−1^ indicated the presence of hydroxy groups in the molecule. The ^1^H NMR spectrum (Table 1) exhibited a one-proton triplet at δ_H_ 6.07 (t, *J* = 2.2 Hz, H-2) and a two-proton doublet at δ_H_ 6.13 (d, *J* = 2.1 Hz, H-4 and H-6) attributable to a symmetrical 1,3,5-trisubstituted aromatic ring. In addition, the presence of a monosubstituted benzene ring was inferred from the signals at δ_H_ 7.29 (dd, *J* = 8.2 and 1.4 Hz, H-2′′ and H-6′′), 7.25 (dd, *J* = 8.0 and 7.4 Hz, H-3′′ and H-5′′) and δ_H_ 7.17 (tt, *J* = 7.2 and 1.5 Hz, H-4′′), while a signal at δ_H_ 5.61 (t, *J* = 7.3 Hz, H-5′) was assigned to the proton of a trisubstituted double bond. The remaining signals of the ^1^H NMR spectrum were due to six methylenes and a methyl group. The 1,3,5-trisubstituted ring was identified as a 5-alkylresorcinol ring from the HMBC correlations of the aromatic protons H-2, H-4, and H-6 with two phenolic carbons at δ_C_ 159.3 (C-1 and C-3) and the HMBC correlation of H-4 and H-6 with the benzylic methylene carbon at δ_C_ 36.9 (C-1′) (Figure 2). The trisubstituted double bond was located at C-5′,C-6′ of the side chain on the basis of the COSY couplings observed from the benzylic methylene protons H-1′ (δ_H_ 2.48) through a sequence of three methylenes up to the olefinic methine (δ_H_ 5.61, H-5′). The HMBC correlation of the protons H-2′′ and H-6′′ (δ_H_ 7.29) of the monosubstituted benzene ring with the olefinic carbon C-6′ indicated that the phenyl ring was linked to C-6′. The presence of a propyl chain also linked to C-6′ was indicated by the presence of a methyl group (δ_H_ 0.86) coupled in the COSY spectrum with two methylene protons at δ_H_ 1.32 (H-8’) which were correlated in the HMBC spectrum with the olefinic carbon C-6′. On the other hand, the NOESY correlations of the olefinic proton H-5′ with the aromatic protons H-2′′ and H-6′′ defined the *E* geometry of the double bond. These data and the remaining NMR correlations led to the proposal of the structure **1** for schizol A.

**Figure 3:**
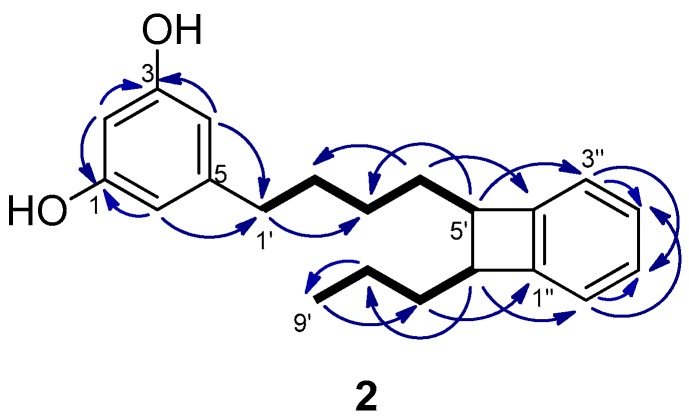
Key COSY (bold bond) and HMBC (→) correlations observed for schizol B (**2**).

The molecular formula of schizol B (**2**), C_21_H_26_O_2_, was established by HRESIMS(−) and indicated that **2** was an isomer of schizol A (**1**). The NMR spectra (Table 1) were related to those of **1**, showing the signals of a resorcinol ring bearing at C-5 an alkyl chain. The signals of a propyl moiety at the end of the chain [δ_H_ 0.97 (3H, t, *J* = 7.4 Hz, H-9′), δ_H_ 1.48 (2H, m, H-8′), and δ_H_ 1.64 (2H, m, H-7′)] were also evident in the spectra of **2**. However, significant differences were observed with respect to the spectra of **1**, in particular the presence of signals due to a disubstituted benzene ring [δ_H_ 6.99 (1H, m, H-3′′), δ_H_ 7.02 (1H, m, H-6′′), and δ_H_ 7.11 (2H, m, H-4′′ and H-5′′)], the absence of the signals due to the double bond at C-5′,C-6′, and the presence of two methines at δ_C_ 51.43 (C-5′)/δ_H_ 2.94 (td, *J* = 7.4 and 2.1 Hz, H-5′) and δ_C_ 51.37 (C-6′)/δ_H_ 2.95 (td, *J* = 7.4 and 2.1 Hz, H-6′). These methines were located at C-5′ and C-6′ of the side chain since the protons at δ_H_ 2.94 (H-5′) and δ_H_ 2.95 (H-6′) were coupled in the COSY spectrum with the methylene protons H-4′ and H-7′, respectively, and in the HMBC with the methylene carbons C-3′ and C-8′, respectively (Figure 3). Moreover, the HMBC correlations of the methine protons H-5′ and H-6′ with the aromatic carbons C-3′′ and C-6′′, respectively, indicated that the methines at C-5′ and C-6′ were linked to adjacent positions of the benzene ring. Further support was obtained from the HMBC correlations of the methylene protons H-7′ and H-4′ with the aromatic carbons C-1′′ and C-2′′, respectively.

Because of the close chemical shifts of H-5′ and H-6′ it was not possible to obtain unambiguous information on the relative configuration at C-5′ and C-6′ from the NOESY spectrum. The spectra were also recorded in CD_3_COCD_3_, C_5_D_5_N, and CD_3_SOCD_3_ but no improvement of the resolution of the signals of the methines at C-5′ and C-6′ was achieved. Interestingly, looking in literature for NMR data of compounds encompassing a similar 1,2-dihydrocyclobutabenzene moiety we found that the chemical shifts of the methines at C-1 and C-2 in *trans*-1,2-diethyl-6,7-dimethyl-1,2- dihydrocyclobuta[a]naphthalene (δ_C_ 51.6 and 51.4, δ_H_ 3.06 and 3.18–3.25) differed significantly from those of the *cis* isomer (δ_C_ 48.5 and 48.4, δ_H_ 3.64–3.72 and 3.51) [14]. The similarity of the chemical shifts of C-5′, C-6′, H-5′, and H-6′ of compound **2** with those reported for the methines of the four membered ring of *trans*-1,2-diethyl-6,7-dimethyl-1,2-dihydrocyclobuta[a]naphthalene supported a *trans* relationship of H-5′ and H-6′ in compound **2**. All these data led to the proposal of the structure **2** for schizol B.

Schizol C (**3**), whose molecular formula C_21_H_26_O_5_S was established by HRESIMS(−), displayed NMR spectra (Table 2) similar to those of schizol A (**1**). Moreover, the COSY and HMBC correlations confirmed that **3** possessed the same carbon framework as **1** and a double bond at C-5′,C-6′ with the *E* geometry ascertained by the NOESY correlation between the methylene protons H-4′ (δ_H_ 2.22) and H-7′ (δ_H_ 2.49). However, the ^1^H NMR spectrum of **2** exhibited three resonances of *meta*-coupled aromatic protons [δ_H_ 6.64 (1H, br dd *J* = 1.8 and 1.5 Hz), δ_H_ 6.60 (1H, dd, *J* = 2.2 and 2.2 Hz), and δ_H_ 6.45 (1H, br dd, *J* = 1.8 and 1.5 Hz)] which defined the 1,3,5-trisubstitution pattern and indicated that the ring was not symmetrically substituted. In particular, the presence of a hydroxy group and a sulfate group on the aromatic ring was deduced from the ^13^C NMR resonances at δ_C_ 159.0 (C-3) and δ_C_ 154.7 (C-1), respectively, together with the molecular formula of **3**, which differed from that of **1** by a SO_3_ unit. The presence of a sulfate group in **3** was also consistent with the IR absorptions at 1239 and 1053 cm^−1^. Moreover, *O*-sulfation of a phenolic hydroxy group has been shown to cause deshielding of protons and carbons at the *ortho* and *para* positions and the shielding of the carbon carrying the sulfate group [15]. Upon comparison with compound **1**, the aromatic protons H-2, H-4, and H-6 of **3** exhibited higher chemical shifts (∆δ_H_ = +0.53, +0.32, and +0.51, respectively) and also the carbons C-2, C-4, and C-6 (∆δ_C_ = +6.1, +5.0, and +5.7 ppm, respectively), while C-1 exhibited a lower chemical shift (∆δ_C_ = −4.6). All these data strongly supported the presence of a sulfate group in the resorcinol ring of schizol C (**3**).

The molecular formula of schizol D (**4**), C_21_H_26_O_5_S, was established by HRESIMS(−). The NMR spectra (Table 2) showed the signals of two methines at δ_C_ 51.44 (C-5′)/δ_H_ 2.94 (1H, td, *J* = 7.7 and 2.0 Hz, H-5′) and δ_C_ 51.37 (C-6′)/δ_H_ 2.95 (1H, td, *J* = 7.7 and 2.0 Hz, H-6′) which indicated the presence in **4** of a 1,2-dihydrocyclobutabenzene moiety similar to that described in compound **2**. On the other hand, the ^13^C NMR signals at δ_C_ 159.0 (C-3) and δ_C_ 154.6 (C-1) were attributable to two aromatic carbons bearing a hydroxy group and a sulfate group, respectively. These and the remaining NMR data led to the conclusion that schizol D (**4**) was the monosulfate derivative of compound **2**. Moreover, the sulfation of the hydroxy group at C-1 was consistent with the higher ^1^H and ^13^C chemical shifts of the aromatic methines C-2, C-4, and C-6 in compound **4** with respect to the same methines in compound **2**.

Schizol E (**5**) possessed the molecular formula C_21_H_26_O_8_S_2,_ that was established by HRESIMS(−). The NMR spectra were related to those of schizol A (**1**) except for the chemical shifts of the protons and carbons of the resorcinol ring (Table 3). In particular, upon comparison with **1**, the signal at δ_C_ 154.2 (C-1 and C-3) in the ^13^C NMR spectrum of **5** was consistent with the *O*-sulfation of the two hydroxy groups of the resorcinol ring. This proposal was supported by the chemical shifts of H-2, H-4, and H-6 [δ_H_ 7.04 (3H, m)] which were significantly higher than the shifts of the same protons in compound **1** (**∆**δ_H_ = +0.97, +0.91, +0.91, respectively). Similarly, the presence of two sulfate groups explains the strong deshielding of carbons C-2 (δ_C_ 113.3), C-4 (δ_C_ 118.8), and C-6 (δ_C_ 118.8) upon comparison with the same carbons in **1** (∆δ_C_ = +12.3, +10.9, +10.9, respectively). These and the remaining spectroscopic data led us to propose the structure **5** for schizol E.

The ^1^H and ^13^C NMR spectra of schizol F (**6**) were similar to those of schizol B (**2**) except for the signals of the resorcinol ring. These data, together with the molecular formula C_21_H_26_O_8_S_2_ established by HRESIMS(−), indicated that **6** was the disulfate derivative of **2**. This proposal was fully confirmed by conversion of **6** into **2** upon acid treatment of **6** that caused the hydrolysis of the sulfate esters. 

From a structural point of view, compounds **1**–**6** obtained from *S. mamillata* represent new types of alkylresorcinol-based natural products. The distinctive feature of compounds **1**, **3**, and **5** is the presence of a phenyl branch on the side chain, an unprecedented characteristic among the 5-alk(en)ylresorcinols previously described in the literature [16]. Most of the known natural products displaying an alk(en)ylresorcinol-based structure have been isolated from several families of higher plants and typically feature a linear side chain with length ranging from C_5_ to C_29_, that is saturated or contains up to four double bonds, and in some instances bearing hydroxy, acetoxy or keto groups. Moreover, although a few 5-alkyl- and 5-alkenylresorcinol derivatives described from plants also contain a phenyl ring, this is always located at the end of the alk(en)yl chain [17,18]. Two species of brown macroalgae and several bacteria and fungi have also been shown to contain simple 5-alkylresorcinols similar to those of terrestrial plants [16,19].

On the other hand, compound **2** and its sulfated derivatives **4** and **6** are characterized by containing a 1,2-dihydrocyclobutabenzene moiety, which is extremely unusual among natural products. To the best of our knowledge the only previous data regard homoisoflavanones of the scillascillin type, first isolated from the plant *Scilla scilloides* Druce [20], and then from other species of the family Hyacinthaceae [21,22,23].

Previous reports on the isolation of alkylresorcinols from marine invertebrates are scarce. The first account described eleven simple alkyl- and alkenylresorcinols, with side chains from C_13_ to C_21_, from a sponge of the genus *Haliclona* [24]. More recently, the study of the bryozoan *Pentapora fascialis* yielded three compounds exhibiting two 5-alkenylresorcinol residues, which contain two double bonds and a sulfate function on a C_15_ side chain, and are linked through a disulfide bridge [13].

Bryozoans are known to host communities of microorganisms and consequently, the hypothesis that many natural products isolated from these invertebrates may be truly produced by associated microorganisms was proposed soon after the first chemical studies of bryozoans [25]. This has been demonstrated for the bryostatins, which are produced by “*Candidatus* Endobugula sertula”, a symbiotic bacterium of the bryozoan *Bugula neritina* [12]. In other instances, such as the tambjamines of *Sessibugula translucens*, the microbial origin is supported by their structural resemblance to a compound isolated from the marine bacterium *Pseudoalteromonas tunicata* [26]. With regard to the origin of compounds **1**–**6** isolated from *S. mamillata*, the possibility that they are actually produced by microorganisms cannot be disregarded. In this context, it is worth noting that recent reports have described the isolation of a series of 2,5-dialkylresorcinol derivatives from marine cyanobacteria [27,28].

### 2.2. Synthesis of Schizol A *(**1**)*

After the isolation of schizols A–F (**1**–**6**), we turned our attention to the synthesis of these metabolites. Herein, we describe a synthetic route for the obtention of schizol A (**1**). Extensive work of synthesis of 5-alkylresorcinols from plants has been reported [16]. The critical step in the synthesis of alkylresorcinols is always the formation of the C-C bond between the aromatic ring and the alkyl side chain. For this purpose, several approaches including Suzuki [29] and Sonogashira [19] couplings or Grignard reagents [30] have been employed. However, the most common procedure entails a Wittig reaction using 3,5-dimethoxybenzaldehyde and the corresponding phosphonium ylide as starting materials [31,32]. Differing from other syntheses of alkylresorcinols, the obtention of schizol A (**1**) posed an additional key step, namely, the formation of the trisubstituted double bond.

**Scheme 1:**
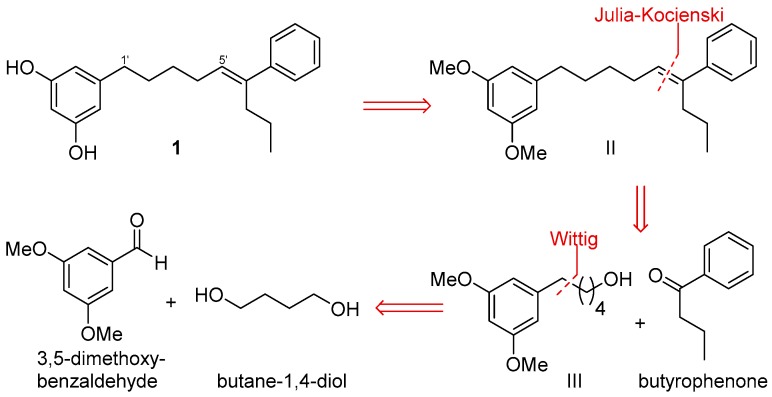
Retrosynthetic analysis of schizol A (**1**).

Scheme 1 outlines a retrosynthetic protocol aimed at the target molecule. The natural product **1** can be prepared from key C-1′/C-2′ and C-5′/C-6′ double-bond disconnections such as Wittig and Julia–Koscienski olefination, respectively, that will allow the formation of the carbon skeleton from the commercially available compounds 3,5-dimethoxybenzaldehyde, butane-1,4-diol, and butyrophenone.

**Scheme 2:**
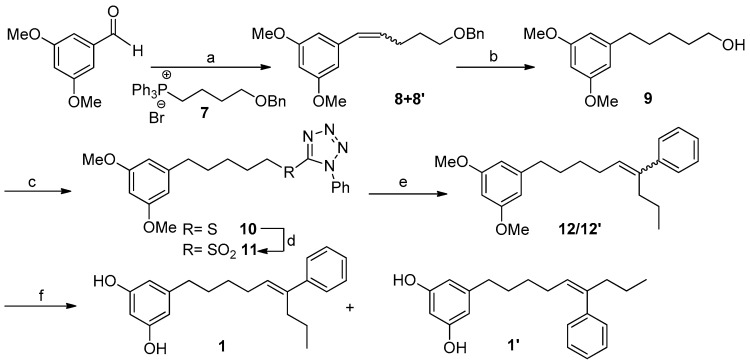
Reagents and conditions: (**a**) LiHMDS, THF, **7**, −78 °C, 2 h, 60%; (**b**) H_2_, Pd/C, AcOEt, 15 h, 96%; (**c**) PPh_3_, DEAD, HSP_T_, 0 °C, THF, 24 h, quant.; (**d**) (NH_4_)_6_Mo_7_·4H_2_O/H_2_O_2_, 30%, EtOH, 0 °C, 20 h, 97%; (**e**) LiHMDS, THF/HMPA (9:1), butyrophenone, −78 °C to rt, 24 h, 32%; (**f**) EtSNa, DMF, ∆, 24 h, 75%.

Our synthetic strategy is shown in Scheme 2. The synthesis starts with the Wittig reaction between 3,5-dimethoxybenzaldehyde and the alkyl phosphonium salt **7**, easily prepared from butane-1,4-diol (see Supplementary Materials pages S7 and S8). This reaction led to a mixture of the isomeric alkene compounds **8**/**8′** (30:70) in 60% yield [33]. This isomer distribution was not of consequence, since arrival at alcohol **9** was founded on subsequent catalytic hydrogenation. Thus, the treatment of **8**/**8′** with 10% Pd-C/H_2_ (1 atm) led almost quantitatively to the removal of the benzyl protecting group and the concomitant alkene reduction, providing the alcohol **9** which accounted for the left-hand fragment of the target molecule **1**. 

**Scheme 3:**
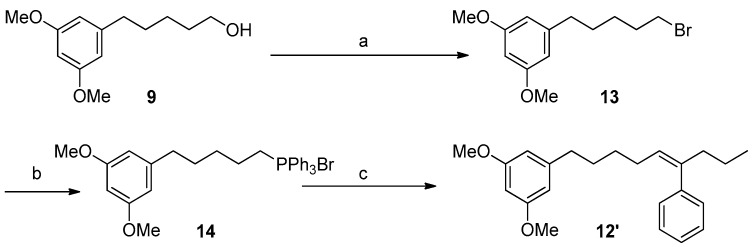
Reagents and conditions: (**a**) PPh_3_, CBr_4_, CH_2_Cl_2_, 0 °C to rt, 2 h, 84%; (**b**) PPh_3_/∆, overnight, 99%; (**c**) LiHMDS, THF, butyrophenone, −78 °C to rt, 2 h, 28%.

At this point, the coupling with butyrophenone would allow us to obtain the complete carbon skeleton of **1**. In particular, the installation of the double bond at C5′,C6′ of **1** was carried out by a Julia–Kocienski olefination [34,35]. Thus, alcohol **9** was treated with 1-phenyl-1*H*-tetrazol-5-thiol in a Mitsunobu reaction yielding in good yield the corresponding sulfide **10**, which was oxidized using (NH_4_)_6_Mo_7_O_24_·4H_2_O and H_2_O_2_ 30% to obtain the olefinating reagent **11** [36,37]. With the sulfone **11** in hand and some experimentation, we defined better conditions for the coupling with butyrophenone, including the preformation of the deprotonated sulfone at −78 °C with LiHMDS in a mixture of THF/HMPA (9:1) and the subsequent addition of an excess of butyrophenone solution in THF. Under these conditions an inseparable mixture (1:4) of the *E*-alkene **12** and its *Z* isomer **12′** was obtained. Although a better *E*-selectivity was expected for this reaction [38] our results are in line with the data recently reported for Julia reactions using aromatic ketones [39]. Alternatively, the formation of the double bond at C5′,C6′ was accomplished by a Wittig reaction that yielded compound **12′** as the only isomer (Scheme 3).

For the *O*-demethylation of compounds **12** and **12′** we first attempted standard Lewis acid conditions such as BBr_3_ in CH_2_Cl_2_ [40] or the treatment with an excess of Py·HCl [41]. Both procedures led, in moderate to good yields, to an *E*/*Z* mixture of the alkene at C-5′,C-6′ (**1** and **1′**) together with some amounts of the *E*/*Z* alkenes at C-6′,C-7′, formed by acid-catalyzed double bond isomerization. To prevent the isomerization of the double bond installed in compounds **12** and **12′** we turned our attention to demethylation procedures under basic conditions. In particular, the treatment of a mixture 1:4 of the alkenes **12**/**12′** with EtSNa in DMF [42] allowed the obtention of the demethylated compounds **1**/**1′** in 75% yield, which were separated by HPLC. The synthetic compound **1** was identical in all respects to the natural metabolite schizol A (**1**).

Therefore, we have achieved the synthesis of the new natural product schizol A (**1**) and its *Z* isomer **1′**. Key synthetic strategies include two C-C bond formation reactions to directly construct the linear skeleton and the route can be easily applied to the synthesis of analogs with different substitution pattern, chain length, or double bond stereochemistry. Furthermore, the synthesis herein described does not need expensive starting materials or reagents nor harsh conditions for most steps.

### 2.3. Antioxidant Activity

We have assessed the antioxidant properties of schizols A–F (**1**–**6**) by using the ABTS assay [43]. The results are shown in Table 4, which also includes the activity of Trolox, a synthetic analogue of α-tocopherol that was measured in the same assay. Schizols A (**1**) and B (**2**) exhibited higher activity than the Trolox standard. Schizols C (**3**) and D (**4**) were less active, displaying activity 34% and 46% that of the Trolox, respectively, and schizols E (**5**) and F (**6**) were inactive. 

Previous reports on the antioxidant activity of 5-alk(en)ylresorcinols have described that 5-*n*-heptadecyl- and 5-*n*-heptadecenylresorcinols prevent Fe^2+^-induced peroxidation of fatty acids and phospholipids in liposomal membranes [44] while 5-*n*-pentadecylresorcinol protect erythrocyte membranes against H_2_O_2_-induced oxidation [45]. In addition, a series of 5-alkylresorcinol homologues possessing linear chains C_15_ to C_23_ have been shown to reduce the oxidative DNA damage induced by H_2_O_2_ on HT29 cells and inhibit the copper-mediated oxidation of human low-density lipoproteins [46]. However, in DPPH radical scavenging assays these long chain 5-*n*-alkylresorcinols were less active than ferulic acid [46] and showed EC_50_ values significantly higher than those of Trolox and tocopherols [47]. On the other hand, 5-(tridec-4′,7′-dienyl)resorcinol has been reported to exhibit significant activity in the DPPH and in the lipid peroxidation assays [48]. Herein we provide data on the activity of the isolated resorcinol derivatives in the ABTS assay, which as preliminary findings indicate the antioxidant properties of schizols A (**1**) and B (**2**). 

## 3. Materials and Methods

### 3.1. General Experimental Procedures

Optical rotations were measured on a Jasco P-2000 polarimeter (Jasco, Easton, MD, USA). UV-Vis data were obtained on a Varian Cary 50Bio (Varian Inc., Palo Alto, CA, USA) or on a VWR UV-1600PC (VWR, Radnor, PA, USA). IR spectra were recorded on a Perkin-Elmer FT-IR Spectrum Two spectrometer (Perkin Elmer, Boston, MA, USA). ^1^H and ^13^C NMR spectra were recorded on an Agilent 600, 500 or 400 spectrometer (Agilent Technologies, Santa Clara, CA, USA) using CD_3_OD or CDCl_3_ as solvent. Chemical shifts were referenced using the corresponding solvent signals (δ_H_ 3.30 and δ_C_ 49.0 for CD_3_OD; δ_H_ 7.26 and δ_C_ 77.0 for CDCl_3_). COSY, HSQC, HMBC, and NOESY experiments were performed using standard Agilent pulse sequences. High resolution mass spectra (HRMS) were obtained on a Waters XEVO G2-S Mass spectrometer (Waters, Milford, MA, USA). All reactions were carried out under an inert nitrogen atmosphere with dry solvents, using anhydrous conditions unless otherwise stated. Dry Et_2_O, CH_2_Cl_2_, toluene, and THF were obtained by passing these solvents through activated alumina columns under argon atmosphere. DMF and MeOH were purchased in anhydre grade. Reagents were purchased at the highest commercial quality and used without further purification. Reaction yields refer to chromatographically and spectroscopically (^1^H NMR) homogeneous material. Column chromatography was carried out on Merck Silica gel 60 (70–230 mesh) (Merck, Darmstadt, Germany). SPE separations were performed on Supelco DSC18 cartridges (500 mg/3 mL) (Supelco, Bellefonte, PA, USA). HPLC separations were performed on a LaChrom-Hitachi apparatus using a differential refractometer RI-71 or a UV detector L-7400 (Merck, Darmstadt, Germany) working at 254 nm. LiChrospher Si-60 (250 × 10 mm, 10 μm) (Merck, Darmstadt, Germany) and Luna Si (2) (250 × 4.6 mm, 5 μm) (Phenomenex, Torrance, CA, USA) columns were used for separations in normal phase (NP-HPLC). LiChrospher 100 RP-18 (250 × 10 mm, 10 μm) (Merck, Darmstadt, Germany) and Kromasil 100-5C18 (250 × 4 mm, 5 μm) (Hichrom, Berkshire, UK) columns were used for separations in reversed phase mode (RP-HPLC). All solvents were of HPLC grade. ABTS (2,2′-azinobis(3-ethylbenzothiazoline-6-sulphonic acid)) diammonium salt and Trolox (6-hydroxy-2,5,7,8-tetramethylchroman-2-carboxylic acid) were purchased from Sigma (St. Louis, MO, USA).

### 3.2. Biological Material

Specimens of *Schizomavella mamillata*, Hincks 1880 (class Gymnolaemata, order Cheilostomatida, family Bitectiporidae) were collected off the coasts of Cadiz, Spain (36°18’6.869” N, 6°8’55.37” W) and immediately frozen. A voucher specimen (B-Sm-0609) is deposited at the Marine Natural Products Laboratory, Faculty of Marine and Environmental Sciences, University of Cadiz, Puerto Real (Cadiz), Spain.

### 3.3. Extraction and Isolation

Frozen samples of *S. mamillata* were extracted with acetone/MeOH (1:1, *v*/*v*, 2.7 L) at room temperature. The solvent was evaporated under reduced pressure and the aqueous residue was extracted with Et_2_O (50 mL × 4) and then with *n*-butanol (100 mL × 2). The Et_2_O layers were combined, dried over MgSO_4_, and evaporated under reduced pressure to yield 441 mg of extract. The *n*-butanol layers were combined and evaporated under reduced pressure to yield 758 mg of extract. The Et_2_O extract was subjected to silica gel column chromatography (18 × 2.5 cm) using as eluents hexanes/Et_2_O (7:3, *v*/*v*, 70 mL), hexanes/Et_2_O (1:1, *v*/*v*, 150 mL), hexanes/Et_2_O (3:7, *v*/*v*, 50 mL), Et_2_O (50 mL), CHCl_3_/MeOH (8:2, *v*/*v*, 50 mL), CHCl_3_/MeOH (7:3, *v*/*v*, 75 mL), CHCl_3_/MeOH (1:1, *v*/*v*, 100 mL), and finally MeOH (50 mL). The *n*-butanol extract was also subjected to column chromatography using as eluents CHCl_3_/MeOH (85:15, *v*/*v*, 300 mL), CHCl_3_/MeOH (8:2, *v*/*v*, 200 mL), CHCl_3_/MeOH (7:3, *v*/*v*, 300 mL), CHCl_3_/MeOH (1:1, *v*/*v*, 200 mL), and finally MeOH (200 mL). The fractions of the Et_2_O extract that had been eluted with hexanes/Et_2_O (3:7, *v*/*v*) and with Et_2_O were combined and evaporated to dryness yielding a mixture (119.1 mg) that was suspended in MeOH/H_2_O (9:1, *v*/*v*, 2 mL) and transferred onto two SPE-C18 cartridges preconditioned with MeOH/H_2_O (9:1, *v*/*v*, 1 mL). Each cartridge was eluted with 10 mL of MeOH/H_2_O (9:1, *v*/*v*). The obtained solution was evaporated to dryness under reduced pressure and subjected again to separation onto two SPE-C18 cartridges as described above using MeOH/H_2_O (8:2, *v*/*v*). The resulting solution was evaporated under reduced pressure yielding a mixture (43.3 mg) that was subjected to repeated RP-HPLC separations using as eluents MeOH/H_2_O (8:2, *v*/*v*) and CH_3_CN/H_2_O (55:45, *v*/*v*), to yield compounds **1** (9.0 mg) and **2** (6.1 mg). The fractions of the Et_2_O extract and of the *n*-butanol extract that had been eluted with CHCl_3_/MeOH (7:3, *v*/*v*) (58.8 mg and 131.2 mg, respectively) were combined, dissolved in MeOH/H_2_O (7:3, *v*/*v*, 3 mL), and transferred onto three SPE-C18 cartridges preconditioned with MeOH/H_2_O (7:3, *v*/*v*, 1 mL). Each cartridge was eluted with 10 mL of MeOH/H_2_O (7:3, *v*/*v*). After evaporation of the solvent, the resulting mixture (159.8 mg) was subjected to repeated RP-HPLC using as eluents MeOH/H_2_O + 0.1% HCOOH (7:3, *v*/*v*), MeOH/H_2_O (6:4, *v*/*v*), and CH_3_CN/H_2_O (4:6, *v*/*v*), to yield compound **3** (17.6 mg), compound **4** (18.3 mg) and a mixture of compounds **5** and **6** (28.6 mg). This mixture proved to be difficult to separate and after NP-HPLC using CHCl_3_/MeOH (8:2, *v*/*v*) and repeated RP-HPLC using MeOH/H_2_O (6:4, *v*/*v*) some amounts of pure compounds **5** (1.0 mg) and **6** (3.8 mg) were obtained.

Schizol A (**1**): colorless oil; UV (MeOH) λ_max_ (log ε) 203 (4.59), 246 (3.56) nm; IR (film) υ_max_ 3365, 2929, 1599, 1462, 1159, 697 cm^−1^; ^1^H NMR (CD_3_OD, 600 MHz) Table 1; ^13^C NMR (CD_3_OD, 150 MHz) Table 1; HRESIMS *m*/*z* 309.1859 [M − H]^−^ (calcd. for C_21_H_25_O_2_, 309.1855).

Schizol B (**2**): colorless oil; ${\left[\alpha \right]}_{D}^{25}$ +32.0 (*c* 0.07, MeOH); UV (MeOH) λ_max_ (log ε) 204 (4.67), 266 (3.41), 272 (3.43) nm; IR (film) υ_max_ 3360, 2925, 1598, 1454, 1149, 995, 741 cm^−1^; ^1^H NMR (CD_3_OD, 600 MHz) Table 1; ^13^C NMR (CD_3_OD, 150 MHz) Table 1; HRESIMS *m*/*z* 309.1861 [M − H]^−^ (calcd. for C_21_H_25_O_2_, 309.1855).

Schizol C (**3**): colorless oil; UV (MeOH) λ_max_ (log ε) 204 (4.78), 243 (3.86) nm; IR (film) υ_max_ 3428, 2929, 1590, 1457, 1239, 1053, 988, 697 cm^−1^; ^1^H NMR (CD_3_OD, 600 MHz) Table 2; ^13^C NMR (CD_3_OD, 150 MHz) Table 2; HRESIMS *m*/*z* 389.1429 [M − H]^−^ (calcd. for C_21_H_25_O_5_S, 389.1423).

Schizol D (**4**): colorless oil; ${\left[\alpha \right]}_{D}^{25}$ +23.7 (*c* 0.09, MeOH); UV (MeOH) λ_max_ (log ε) 202 (4.52), 265 (3.32), 272 (3.37) nm; IR (film) υ_max_ 3443, 2925, 1591, 1455, 1240, 1054, 989, 740 cm^−1^; ^1^H NMR (CD_3_OD, 600 MHz) Table 2; ^13^C NMR (CD_3_OD, 150 MHz) Table 2; HRESIMS *m*/*z* 389.1424 [M − H]^−^ (calcd. for C_21_H_25_O_5_S, 389.1423).

Schizol E (**5**): colorless oil; UV (MeOH) λ_max_ (log ε) 202 (4.68), 245 (3.19) nm; IR (film) υ_max_ 3445, 2927, 1614, 1591, 1454, 1236, 1058, 981 cm^−1^; ^1^H NMR (CD_3_OD, 600 MHz) Table 3; ^13^C NMR (CD_3_OD, 150 MHz) Table 3; HRESIMS *m*/*z* 491.0794 [M − 2H + Na]^−^ (calcd. for C_21_H_24_O_8_S_2_Na, 491.0810).

Schizol F (**6**): colorless oil; ${\left[\alpha \right]}_{D}^{25}$ +16.2 (*c* 0.09, MeOH); UV (MeOH) λ_max_ (log ε) 202 (4.68) nm; IR (film) υ_max_ 3445, 2925, 1615, 1590, 1455, 1236, 1061, 981 cm^−1^; ^1^H NMR (CD_3_OD, 600 MHz) Table 3; ^13^C NMR (CD_3_OD, 150 MHz) Table 3; HRESIMS *m*/*z* 491.0814 [M − 2H + Na]^−^ (calcd. for C_21_H_24_O_8_S_2_Na, 491.0810).

### 3.4. Hydrolysis of Schizol F *(**6**)*

Compound **6** (2.8 mg) was dissolved in HC1 1 M (1 mL) and heated at 90 °C for 5 min. After cooling at rt, the pH was adjusted to 5 with NaOH 1 M and the solution was extracted with EtOAc (2 × 2 mL). The organic layers were combined, washed with brine (2 mL), dried over anhydrous MgSO_4_ and the solvent evaporated. The residue was purified by HPLC (MeOH/H_2_O 8:2, *v*/*v*) to yield compound **2** (1.0 mg).

### 3.5. Synthesis of Schizol A *(**1**)*

#### 3.5.1. Synthesis of Compounds **8**/**8′**

A flame dried flask equipped with a stir bar under nitrogen atmosphere was charged with a solution of 560 mg of **7** (1.11 mmol) in 5 mL of dry THF and 1.5 mL of LiN(SiMe_3_)_2_ 1 M in THF under an atmosphere of nitrogen. After 30′ of stirring at rt, the mixture was cooled to −78 °C and 1.0 mmol of 3,5-dimethoxybenzaldehyde in 1 mL of THF was added dropwise. After 2 h at rt the reaction was quenched with 5 mL of a saturated solution of NH_4_Cl and extracted with EtOAc (3 × 5 mL). The organic phase was dried over anhydrous MgSO_4_ and the solvent concentrated in vacuo yielding an oily residue that was purified by CC (hexanes/Et_2_O 9:1) to yield compounds **8/8′** (187 mg, 0.60 mmol, 60%). For the characterization of these alkenes, a mixture of **8/8′** was subjected to HPLC (hexanes/EtOAc 98:2).

Compound **8** (isomer *E*): IR (film) υ_max_ 3030, 2936, 2839, 1591, 1455, 1204, 1151, 736, 697 cm^−1^; ^1^H NMR (400 MHz, CDCl_3_) δ 7.36–7.26 (m, 5H, H3′′-H7′′), 6.50 (d, *J* = 2.4 Hz, 2H, H2′ and H6′), 6.33 (d, *J* = 15.8 Hz, 1H, H1), 6.21 (dt, *J* = 15.8, 6.7 Hz, 1H, H2), 6.29 (t, *J* = 2.2 Hz, 1H, H4′), 4.52 (s, 2H, H1′′), 3.80 (s, 6H, -OMe), 3.53 (t, *J* = 6.4 Hz, 2H, H5), 2.32 (m, 2H, H3), 1.81 (quint., *J* = 6.5 Hz, 2H, H4); ^13^C NMR (see Supplementary Materials page S9); HRESIMS *m*/*z* 313.1805 [M + H]^+^ (calcd. for C_20_H_25_O_3_, 313.1804). 

Compound **8′** (isomer *Z*): IR (film) υ_max_ 3004, 2935, 2854, 1590, 1454, 1204, 1155, 736, 697 cm^−1^; ^1^H NMR (400 MHz, CDCl_3_) δ 7.33 (m, 2H, H4′′ and H6′′), 7.30 (m, 2H, H3′′ and H7′′), 7.28 (m, 1H, H5′′), 6.46 (d, *J* = 2.1 Hz, 2H, H2′ and H6′), 6.39 (d, *J* = 11.7 Hz, 1H, H1), 6.38 (t, *J* = 2.1 Hz, 1H, H4′), 5.68 (dt, *J* = 11.7, 7.3 Hz, 1H, H2), 4.49 (s, 2H, H1′′), 3.78 (s, 6H, -OMe), 3.52 (t, *J* = 6.5 Hz, 2H, H5), 2.47 (m, 2H, H3), 1.79 (quint., *J* = 6.7 Hz, 2H, H4); ^13^C NMR (see Supplementary Materials page S9); HRESIMS *m*/*z* 313.1812 [M + H]^+^ (calcd. for C_20_H_25_O_3_, 313.1804).

#### 3.5.2. Synthesis of Compound **9**

A total of 62 mg of Pd/C was added to alkenes **8/8′** (2 mmol) dissolved in 5 mL of EtOAc. The reaction mixture was subjected to a hydrogen atmosphere (1 atm) for 15 h. The catalyst was removed by filtration and the resulting solution was taken to dryness yielding 430 mg (1.92 mmol, 96%) of compound **9** as a colorless oil.

Compound **9**: IR (film) υ_max_ 3359, 3026, 2935, 2858, 1596, 1462, 1205, 1150, 1057, 829, 695 cm^−1^; ^1^H NMR (400 MHz, CDCl_3_) δ 6.33 (d, *J* = 2.2 Hz, 2H, H2′ and H6′), 6.29 (t, *J* = 2.2 Hz, 1H, H4′), 3.76 (s, 6H, -OMe), 3.61 (t, *J* = 6.6 Hz, 2H, H1), 2.55 (t, *J* = 7.7 Hz, 2H, H5), 1.63 (m, 2H, H4), 1.58 (m, 2H, H2), 1.39 (m, 2H, H3); ^13^C NMR (see Supplementary Materials page S9); HRESIMS *m*/*z* 225.1502 [M + H]^+^ (calcd. for C_13_H_21_O_3_: 225.1491).

#### 3.5.3. Synthesis of Compound **10**

To 200 mg of **9** (0,89 mmol) in 10 mL of dry THF and under an inert atmosphere, 280 mg of PPh_3_ (1.07 mmol) and 239 mg of 1-phenyl-1*H*-tetrazol-5-thiol (1.34 mmol) were added at rt. The reaction mixture was cooled to 0 °C with an ice water bath and diethyl azodicarboxylate (DEAD) 40% in toluene (520 µL, 1.16 mmol) was added. After 24h at rt, the solvent was evaporated and the residue purified by CC (hexanes/EtOAc (9:1 → 75:25)), yielding **10** quantitatively.

Compound **10**: IR (film) υ_max_ 2932, 2855, 1594, 1460, 1427, 1203, 1149, 760, 693 cm^−1^; ^1^H NMR (400 MHz, CDCl_3_) δ 7.58–7.52 (m, 5H, Ph), 6.33 (d, *J* = 2.3 Hz, 2H, H2′ and H6′), 6.29 (t, *J* = 2.3 Hz, 1H, H4′), 3.77 (s, 6H, -OMe), 3.38 (t, *J* = 7.4 Hz, 2H, H1), 2.56 (t, *J* = 7.6 Hz, 2H, H5), 1.86 (quint, *J* = 7.5 Hz, 2H, H2), 1.66 (m, 2H, H4), 1.49 (m, 2H, H3); ^13^C NMR (see Supplementary Materials page S9); HRESIMS *m*/*z* 385.1703 [M + H]^+^ (calcd. for C_20_H_25_SO_2_N_4_, 385.1698).

#### 3.5.4. Synthesis of Compound **11**

To a solution of **10** (277 mg, 0.72 mmol) in 10 mL of EtOH at 0 °C a mixture of (NH_4_)_6_Mo_7_O_24_·4H_2_O (89 mg, 0.072 mmol) and H_2_O_2_ 30% (0.85 mL, 8.5 mmol) was added. After stirring for 20 h at rt, the reaction mixture was quenched by addition of H_2_O (10 mL) and extracted with CH_2_Cl_2_ (3 × 20 mL). The organic layers were separated, dried over anhydrous MgSO_4,_ and the solvent evaporated at reduced pressure to yield 292 mg (0.70 mmoles, 97%) of **11**.

Compound **11**: IR (film) υ_max_ 2925, 2853, 1594, 1460, 1340, 1150, 1050, 762, 689 cm^−1^; ^1^H NMR (400 MHz, CDCl_3_) δ 7.70–7.57 (m, 5H, Ph), 6.32 (d, *J* = 2.3 Hz, 2H, H2′ and H6′), 6.31 (t, *J* = 2.3 Hz, 1H, H4′), 3.78 (s, 6H, -OMe), 3.72 (m, 2H, H1), 2.57 (t, *J* = 7.6 Hz, 2H, H5), 1.98 (m, 2H, H2), 1.66 (m, 2H, H4), 1.53 (m, 2H, H3); ^13^C NMR (see Supplementary Materials page S9); HRESIMS *m*/*z* 417.1604 [M + H]^+^ (calcd. for C_20_H_25_SO_4_N_4_: 417.1597).

#### 3.5.5. Synthesis of Compounds **12**/**12′** (Julia–Kocienski Reaction)

A total of 95 mg of **11** (0.228 mmol) was disolved in 3 mL of THF/HMPA 9:1 under nitrogen atmosphere. The solution was cooled to −78 °C and LiHMDS 1M in THF (350 µL, 0.350 mmol) was added. After 30’ at −78 °C, butyrophenone (50 µL, 0.350 mmol) in 1 mL of THF was added and the reaction was stirred for another 30’ at this temperature. Then, the reaction was stirred for 24 h at rt, quenched with NH_4_Cl (10 mL), and extracted with EtOAc (3 × 20 mL). The combined organic phases were washed with brine (2 × 20 mL) and dried over anhydrous MgSO_4_. The solvent was evaporated under reduced pressure and the residue purified by CC (hexanes/Et_2_O, 98:2) to yield 25 mg of an inseparable mixture of **12/12′** (32%) in a 1:4 ratio that was used in the next reaction step.

#### 3.5.6. Synthesis of Compounds **1**/**1′**

To 75 mg of EtSNa (0.092 mmol) under nitrogen atmosphere and stirring, a solution of **12**/**12′** (0.089 mmol) in 1 mL of DMF was added. The reaction mixture was refluxed for 24 h. After cooling to 0 °C, a 5% aqueous HCl solution was added, the aqueous layer was extracted with EtOAc, and the combined organic layers were dried over anhydrous MgSO_4_ and evaporated in vacuo. The residue was purified by CC (hexanes/Et_2_O, 3:7) to yield 21 mg of a mixture of **1/1′** (75%) that was subjected to HPLC in reversed phase mode (CH_3_CN/H_2_O, 55:45) to yield pure **1** (7 mg, 0.023 mmol, 25%) and **1′** (14 mg, 0.045 mmol, 49%). Compound **1** was identical in all respects to the natural schizol A.

Compound **1′:** IR (film) υ_max_ 3348, 3077, 2956, 2929, 2857, 1598, 1492, 1462, 1303, 1150, 700 cm^−1^; ^1^H NMR (600 MHz, CDCl_3_) δ 7.30 (brt, *J* = 7.6 Hz, 2H, H3′′ and H5′′), 7.09 (dd, *J* = 8.1, 1.2 Hz, 2H, H2′′ and H6′′), 7.21 (tt, *J* = 7.4, 1.3 Hz, 1H, H4′′), 6.06 (brs, 3H, H2, H4 and H6), 5.41 (t, *J* = 7.4 Hz, 1H, H5′), 2.33 (t, *J* = 7.7, 2H, H1′), 2.30 (t, *J* = 7.5, 2H, H7′), 1.92 (td, *J* = 7.4, 7.4, 2H, H4′), 1.47 (quint, *J* = 7.6, 2H, H2′), 1.33 (quint, *J* = 7.5, 2H, H3′), 1.28 (sext, *J* = 7.4, 2H, H8′), 0.85 (t, *J* = 7.4 Hz, 3H, H9′); ^13^C NMR (see Supplementary Materials page S10); HRESIMS *m*/*z* 311.2018 [M + H]^+^ (calcd. for C_21_H_27_O_2_, 311.2011).

#### 3.5.7. Synthesis of Compound **13**

To a solution of 1.12 g of **9** (5.0 mmol) and 1.57 g of PPh_3_ (6.0 mmol) in 15 mL of CH_2_Cl_2_ at 0 °C, 1.82 g of CBr_4_ (15.5 mmol) was added. The resulting mixture was stirred at rt for 2 h and then concentrated under reduced pressure to give a residue that was purified by CC (hexanes/Et_2_O 9:1) to yield Compound **13** (1.19 g, 4.20 mmol, 84%) as a colorless oil.

Compound **13**: ^1^H NMR (400 MHz, CDCl_3_) δ 6.35 (d, *J* = 2.3 Hz, 2H, H2′ and H6′), 6.31 (t, *J* = 2.3 Hz, 1H, H4′), 3.79 (s, 6H, -OMe), 3.41 (t, *J* = 7.0 Hz, 2H, H1), 2.58 (t, *J* = 7.3 Hz, 2H, H5), 1.89 (m, 2H, H2), 1.65 (m, 2H, H4), 1.49 (m, 2H, H3); ^13^C NMR (see Supplementary Materials page S10); HRESIMS *m*/*z* 287.0680 [M + H]^+^ (calcd. for C_13_H_20_O_2_^79^Br, 287.06474), *m*/*z* 289.0645 [M + H]^+^ (calcd. for C_13_H_20_O_2_^81^Br, 289.0626).

#### 3.5.8. Synthesis of Compound **14**

A total of 288 mg of **13** (1.00 mmol) and 262 mg of PPh_3_ (1.00 mmol) was heated overnight in an oven at 100 °C yielding 542 mg of **14** (0.99 mmol, 99%) as an amorphous white solid.

Compound **14**: ^1^H NMR (400 MHz, CDCl_3_) δ 7.79 (m, 6H, H2′′ and H6′′), 7.75 (m, 3H, H4′′), 7.66 (m, 6H, H3′′ and H5′′), 6.23 (m, 2H, H2′ and H6′), 6.22 (m, 1H, H4′), 3.71 (s, 6H, OMe), 3.70 (m, 2H, H1), 2.46 (t, *J* = 7.3 Hz, 2H, H5), 1.64 (m, 2H, H3), 1.58 (m, 2H, H2), 1.56 (m, 2H, H4); ^13^C NMR (see Supplementary Materials page S10); HRESIMS *m*/*z* 469.2307 [M − Br]^+^ (calcd. for C_31_H_34_O_2_P, 469.2296).

#### 3.5.9. Synthesis of Compound **12′** (Wittig Reaction)

A flame dried flask equiped with a stir bar under nitrogen atmosphere was charged with a suspension of **14** (1.0 mmol) in 5 mL of dry THF and 1.5 mL of LiN(SiMe_3_)_2_ 1M in THF under an atmosphere of nitrogen. After 30’ of stirring at rt, the mixture was cooled to −78 °C and 1.0 mmol of butyrophenone in 1 mL of THF was added dropwise. After 2 h at rt, the reaction was quenched with 5 mL of a saturated solution of NH_4_Cl and extracted with EtOAc (3 × 5 mL). The organic phase was dried under anhydrous MgSO_4_ and the solvent concentrated in vacuo yielding an oily residue that was purified by CC (hexanes/Et_2_O 95:5 → 1:1) to yield compound **12′** as a colorless oil (94 mg, 0.28 mmol, 28%).

Compound **12′**: IR (film) υ_max_ 3056, 2956, 2933, 2859, 1596, 1461, 1205, 1150, 830, 701 cm^−1^; ^1^H NMR (400 MHz, CDCl_3_) δ 7.33 (tt, *J* = 7.3, 1.2 Hz, 2H, H3′′ and H5′′), 7.24 (tt, *J* = 7.3, 1.2 Hz, 1H, H4′′), 7.14 (m, 2H, H2′′ and H6′′), 6.32 (d, *J* = 1.8 Hz, 2H, H4 and H6), 6.31 (t, *J* = 1.8 Hz, 1H, H2), 5.43 (t, *J* = 7.3 Hz, 1H, H5′), 3.78 (s, 6H, -OCH_3_), 2.49 (t, *J* = 7.6 Hz, 2H, H1′), 2.32 (t, *J* = 7.3 Hz, 2H, H7′),1.98 (m, 2H, H4′), 1.57 (m, 2H, H2′), 1.38 (m, 2H, H3′), 1.33 (sext, *J* = 7.3, 2H, H8′), 0.88 (t, *J* = 7.3 Hz, 3H, H9′); ^13^C NMR (see Supplementary Materials page S10); HRESIMS *m*/*z* 339.2326 [M + H]^+^ (calcd. for C_23_H_31_O_2_, 339.2324).

### 3.6. Antioxidant Assay

Antioxidant activity was determined by the ABTS free radical decolorization assay developed by Re et al*.* [42], with slight modifications. In brief, a solution of the radical cation ABTS^+•^ was prepared by mixing (1:1, *v*/*v*) a solution of ABTS diammonium salt (7 mM) and a solution of potassium persulfate (2.45 mM) in H_2_O. The mixture was kept in the dark at room temperature for 12–18 h before use. Then the solution was diluted with EtOH to an absorbance of 0.70 ± 0.02 at 734 nm. Stock solutions of Trolox (standard) and of the tested compounds were prepared in EtOH. For the assay, 100 μL of the Trolox solution or 100 μL of tested compound solution were mixed with 2 mL of the ABTS^+•^ solution and the absorbance at 734 nm was measured after 6 min. Controls were prepared by adding 100 μL of EtOH to 2 mL of ABTS^+•^ solution. All of the determinations were carried out in triplicate. The percentage of inhibition of the absorbance was calculated by the following equation: % Inhibition = [(A_0_ − A_1_)/A_0_] × 100, where A_0_ expresses the absorbance of control and A_1_ the absorbance of the tested compound.

## 4. Conclusions

Marine organisms continue to be a valuable source of bioactive natural products with unique structural features. In this study of the bryozoan *S. mamillata* we have isolated two new types of alkylresorcinol-derived metabolites, characterized by possessing either a phenyl branch on the side chain (compounds **1**, **3**, and **5**) or a 1,2-dihydrocyclobutabenzene moiety (compounds **2**, **4**, and **6**), the latter structural feature being very rarely found among natural products. Within each group, the isolated compounds differed by the sulfation degree of the phenolic hydroxy groups. We have achieved the synthesis of schizol A (**1**) and its *Z* isomer **1′** by a synthetic strategy that includes two olefination reactions to directly construct the carbon skeleton. Schizols A (**1**) and B (**2**) have shown activity in preliminary antioxidant assays and further studies in this and other bioactivity fields would be of interest. In this context, the synthetic route developed for schizol A (**1**) is flexible enough to access analogs with different substitution patterns, chain lengths, or double bond stereochemistry that will allow us to advance the study of the biological properties of this type of phenolic compounds.

## Figures and Tables

**Table 1:** ^1^H and ^13^C NMR data of schizol A (**1**) and schizol B (**2**) ^a,b^.

Position	1		2	
	δ_C_, Type	δ_H_, m (*J* in Hz)	δ_C_, Type	δ_H_, m (*J* in Hz)
1	159.3, C		159.3, C	
2	101.0, CH	6.07, t (2.2)	101.0, CH	6.08, t (2.2)
3	159.3, C		159.3, C	
4	107.9, CH	6.13, d (2.1)	108.0, CH	6.14, d (2.2)
5	146.2, C		146.2, C	
6	107.9, CH	6.13, d (2.1)	108.0, CH	6.14, d (2.2)
1′	36.9, CH_2_	2.48, t (7.6)	36.9, CH_2_	2.45, t (7.5)
2′	32.1, CH_2_	1.64, m	32.5, CH_2_	1.64, m
3′	30.6, CH_2_	1.49, tt (7.7, 7.4)	29.1, CH_2_	1.52, m
4′	29.4, CH_2_	2.21, dt (7.4, 7.4)	35.1, CH_2_	1.69, m
5′	129.9, CH	5.61, t (7.3)	51.43, CH	2.94, td (7.4, 2.1)
6′	141.6, C		51.37, CH	2.95, td (7.4, 2.1)
7′	32.6, CH_2_	2.48, t (7.5)	37.6, CH_2_	1.64, m
8′	22.8, CH_2_	1.32, tq (7.5, 7.5)	22.8, CH_2_	1.48, m
9′	14.2, CH_3_	0.86, t (7.4)	14.6, CH_3_	0.97, t (7.4)
1′′	144.8, C		148.99 ^c^, C	
2′′	127.4, CH	7.29, dd (8.2, 1.4)	149.05 ^c^, C	
3′′	129.2, CH	7.25, dd (8.0, 7.4)	123.13 ^d^, CH	6.99, m
4′′	127.5, CH	7.17, tt (7.2, 1.5)	127.9, CH	7.11, m
5′′	129.2, CH	7.25, dd (8.0, 7.4)	127.9, CH	7.11, m
6′′	127.4, CH	7.29, dd (8.2, 1.4)	123.07 ^d^, CH	7.02, m

^a^ ^1^H at 600 MHz, ^13^C at 150 MHz; ^b^ Assignments aided by COSY, HSQC, HMBC, and NOESY experiments; ^c,d^ Assignments marked with the same letter in the same column may be interchanged.

**Table 2:** ^1^H and ^13^C NMR data of schizol C (**3**) and schizol D (**4**) ^a,b^.

Position	3		4	
	δ_C_, Type	δ_H_, m (*J* in Hz)	δ_C_, Type	δ_H_, m (*J* in Hz)
1	154.7, C		154.6, C	
2	107.1, CH	6.60, dd (2.2, 2.2)	107.1, CH	6.60, dd (2.4, 2.0)
3	159.0, C		159.0, C	
4	112.9, CH_2_	6.45, br dd (1.8, 1.5)	113.0, CH_2_	6.45, br dd (1.6, 1.6)
5	145.8, C		145.9, C	
6	113.6, CH	6.64, br dd (1.8, 1.5)	113.6, CH	6.63, br dd (1.6, 1.6)
1′	36.8, CH_2_	2.55, t (7.7)	36.9, CH_2_	2.54, (t, 7.5)
2′	32.1, CH_2_	1.66, m	32.5, CH_2_	1.66, m
3′	30.6, CH_2_	1.48, tt (7.7, 7.3)	29.2, CH_2_	1.52, m
4′	29.4, CH_2_	2.22, dt (7.3, 7.3)	35.1, CH_2_	1.69, m
5′	129.9, CH	5.61, t (7.3)	51.44, CH	2.94, td (7.7, 2.0)
6′	141.6, C		51.37, CH	2.95, td (7.7, 2.0)
7′	32.6, CH_2_	2.49, t (7.5)	37.6, CH_2_	1.64, m
8′	22.8, CH_2_	1.33, tq (7.7, 7.3)	22.8, CH_2_	1.49, m
9′	14.2, CH_3_	0.86, t (7.3)	14.6, CH_3_	0.97, t (7.5)
1′′	144.7, C		149.01 ^c^, C	
2′′	127.4, CH	7.29, dd (8.1, 1.3)	149.06 ^c^, C	
3′′	129.2, CH	7.26, dd (8.1, 7.3)	123.18 ^d^, CH	7.01, m
4′′	127.4, CH	7.16, tt (7.3, 1.3)	127.95 ^e^, CH	7.10, m
5′′	129.2, CH	7.26, dd (8.1, 7.3)	127.92 ^e^, CH	7.10, m
6′′	127.4, CH	7.29, dd (8.1, 1.3)	123.07 ^d^, CH	7.01, m

^a 1^H at 600 MHz, ^13^C at 150 MHz; ^b^ Assignments aided by COSY, HSQC, HMBC, and NOESY experiments; ^c–e^ Assignments marked with the same letter in the same column may be interchanged.

**Table 3:** ^1^H and ^13^C NMR data of schizol E (**5**) and schizol F (**6**) ^a,b^.

Position	5		6	
	δ_C_, Type	δ_H_, m (*J* in Hz)	δ_C_, Type	δ_H_, m (*J* in Hz)
1	154.2, C		154.0, C	
2	113.3, CH	7.04, m	113.5, CH	7.11, m
3	154.2, C		154.0, C	
4	118.8, CH	7.04, m	119.1, CH_2_	7.01, br s
5	145.6, C		145.8, C	
6	118.8, CH	7.04, m	119.1, CH	7.01, br s
1′	36.8, CH_2_	2.64, t (7.7)	36.8, CH_2_	2.64, t (7.7)
2′	32.1, CH_2_	1.70, m	32.5, CH_2_	1.69, m
3′	30.7, CH_2_	1.51, m	29.3, CH_2_	1.54, m
4′	29.4, CH_2_	2.24, dt (7.5, 7.3)	35.1, CH_2_	1.71, m
5′	129.9, CH	5.62, t (7.3)	51.44, CH	2.95, br t (7.3)
6′	141.6, C		51.37, CH	2.97, br t (7.3)
7′	32.6, CH_2_	2.50, t (7.7)	37.6, CH_2_	1.65, m
8′	22.8, CH_2_	1.32, m	22.8, CH_2_	1.50, m
9′	14.2, CH_3_	0.86, t (7.4)	14.6, CH_3_	0.98, t (7.4)
1′′	144.8, C		149.0 ^c^, C	
2′′	127.5, CH	7.30, dd (8.1, 1.2)	149.1 ^c^, C	
3′′	129.1, CH	7.25, dd (8.1, 7.5)	123.2 ^d^, CH	7.02, m
4′′	127.4, CH	7.16, m	128.0 ^e^, CH	7.11, m
5′′	129.1, CH	7.25, dd (8.1, 7.5)	127.9 ^e^, CH	7.11, m
6′′	127.5, CH	7.30, dd (8.1, 1.2)	123.1 ^d^, CH	7.02, m

^a 1^H at 600 MHz, ^13^C at 150 MHz; ^b^ Assignments aided by COSY, HSQC, HMBC, and NOESY experiments; ^c–e^ Assignments marked with the same letter in the same column may be interchanged.

**Table 4:** Antioxidant activities of compounds **1**–**4** in the ABTS assay ^a^.

Compound	Trolox	1	2	3	4
EC_50_ (μM ± SD, *n* = 3)	10.43 ± 0.06	6.24 ± 0.03	7.66 ± 0.24	30.25 ± 0.10	22.59 ± 0.53

^a^ ABTS: 2,2′-azinobis(3-ethylbenzothiazoline-6-sulphonic acid).

## Supplementary Materials

The following are available online at www.mdpi.com/1660-3397/15/11/344/s1: Figure S1: ^1^H and ^13^C NMR spectra of **1**; Figure S2: ^1^H and ^13^C NMR spectra of **2**; Figure S3: ^1^H and ^13^C NMR spectra of **3**; Figure S4: ^1^H and ^13^C NMR spectra of **4**; Figure S5: ^1^H and ^13^C NMR spectra of **5**; Figure S6: ^1^H and ^13^C NMR spectra of **6**; Pages S7 and S8: Full experimental details for the chemical synthesis of **7**; Page S9: ^13^C NMR data of **8**, **8’**, **9**, **10** and **11**; Page S10: ^13^C NMR data of **1’**, **12’**, **13**, and **14**.
