# Supplementary material for: 5-Alkylresorcinol Derivatives from the Bryozoan Schizomavella mamillata: Isolation, Synthesis, and Antioxidant Activity
Source: Mar Drugs. 2017 Nov 2;15(11):344. doi: 10.3390/md15110344 (PMC5706034; doi:10.3390/md15110344)
Supplement: Supplementary file 1 [file marinedrugs-15-00344-s001.pdf]

SUPPLEMENTARY MATERIAL

5-Alkylresorcinol Derivatives from the Bryozoan *Schizomavella mamillata*: Isolation, Synthesis, and Antioxidant activity

María J. Ortega, Juan J. Pantoja, Carolina de los Reyes and Eva Zubía[*]

Departamento de Química Orgánica, Facultad de Ciencias del Mar y Ambientales, Universidad de Cádiz, 11510 Puerto Real (Cádiz), Spain

**Figure S1**	^{1}H and ^{13}C NMR spectra of schizol A (**1**)
**Figure S2**	^{1}H and ^{13}C NMR spectra of schizol B (**2**)
**Figure S3**	^{1}H and ^{13}C NMR spectra of schizol C (**3**)
**Figure S4**	^{1}H and ^{13}C NMR spectra of schizol D (**4**)
**Figure S5**	^{1}H and ^{13}C NMR spectra of schizol E (**5**)
**Figure S6**	^{1}H and ^{13}C NMR spectra of schizol F (**6**)
**Pages S7 and S8**	Synthesis of compound **7**
**Page S9**	^{13}C NMR data of compounds **8**, **8′**, **9**, **10** and **11**
**Page S10**	^{13}C NMR data of compounds **1′**, **13**, **14** and **12′**

Figure S1

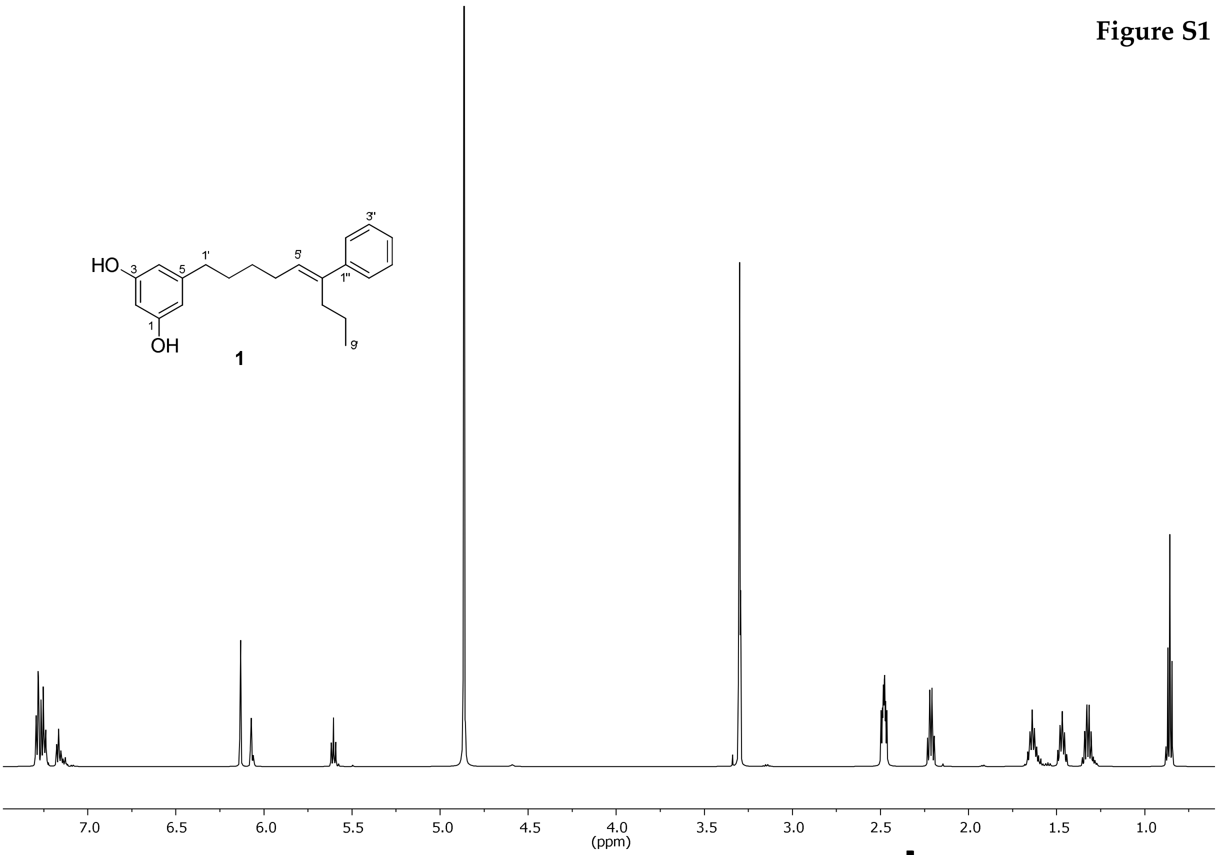

^{1}H NMR spectrum (CD_3OD, 600 MHz) of schizol A (**1**)

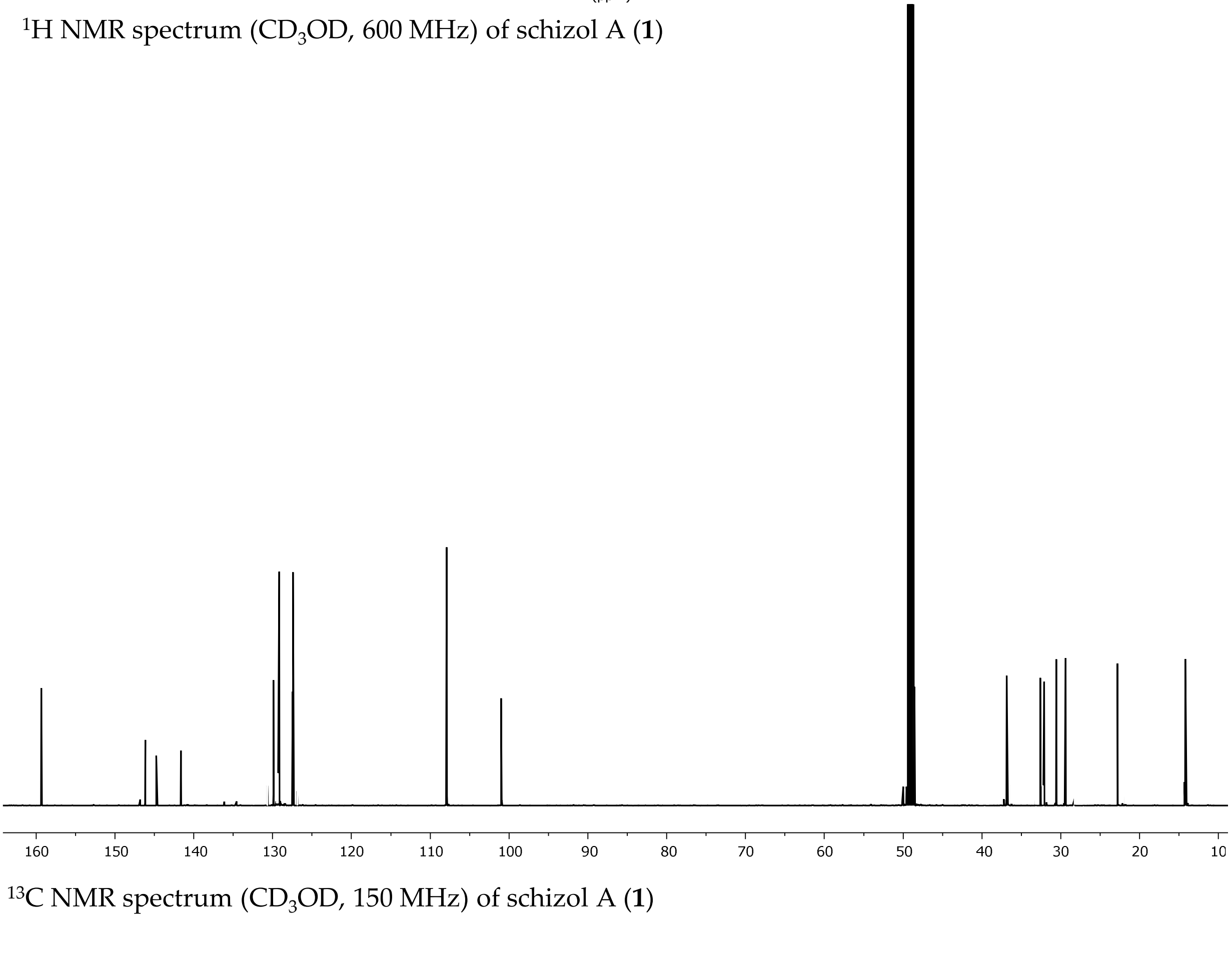

^{13}C NMR spectrum (CD_3OD, 150 MHz) of schizol A (**1**)

Figure S2

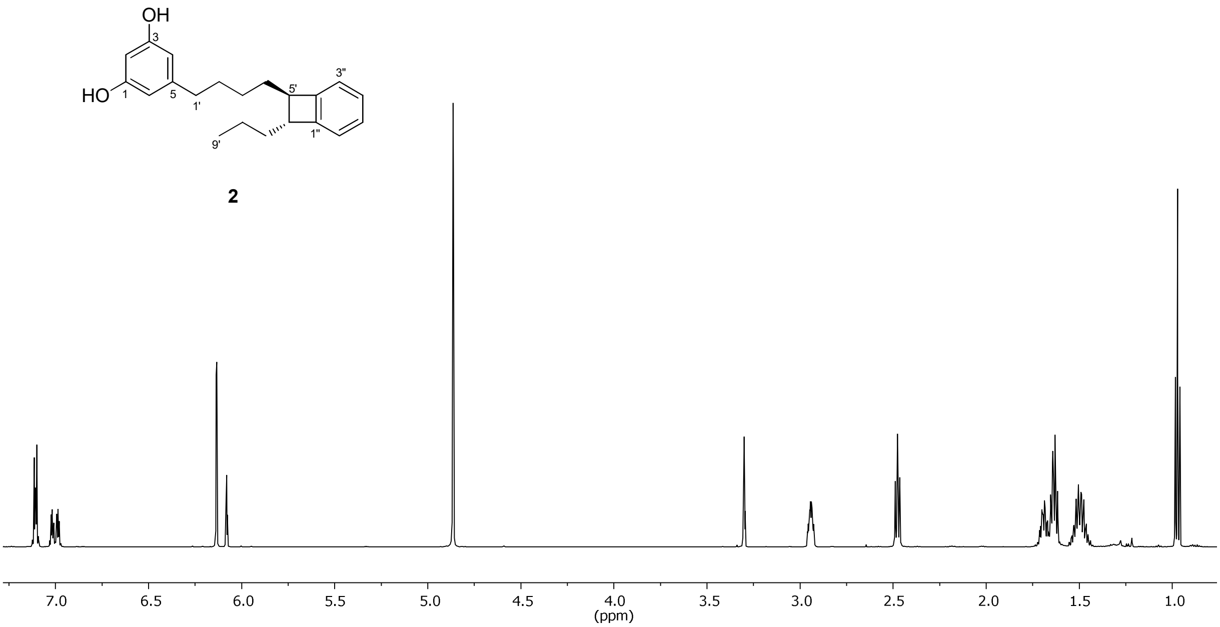

^{1}H NMR spectrum (CD_3OD, 600 MHz) of schizol B (**2**)

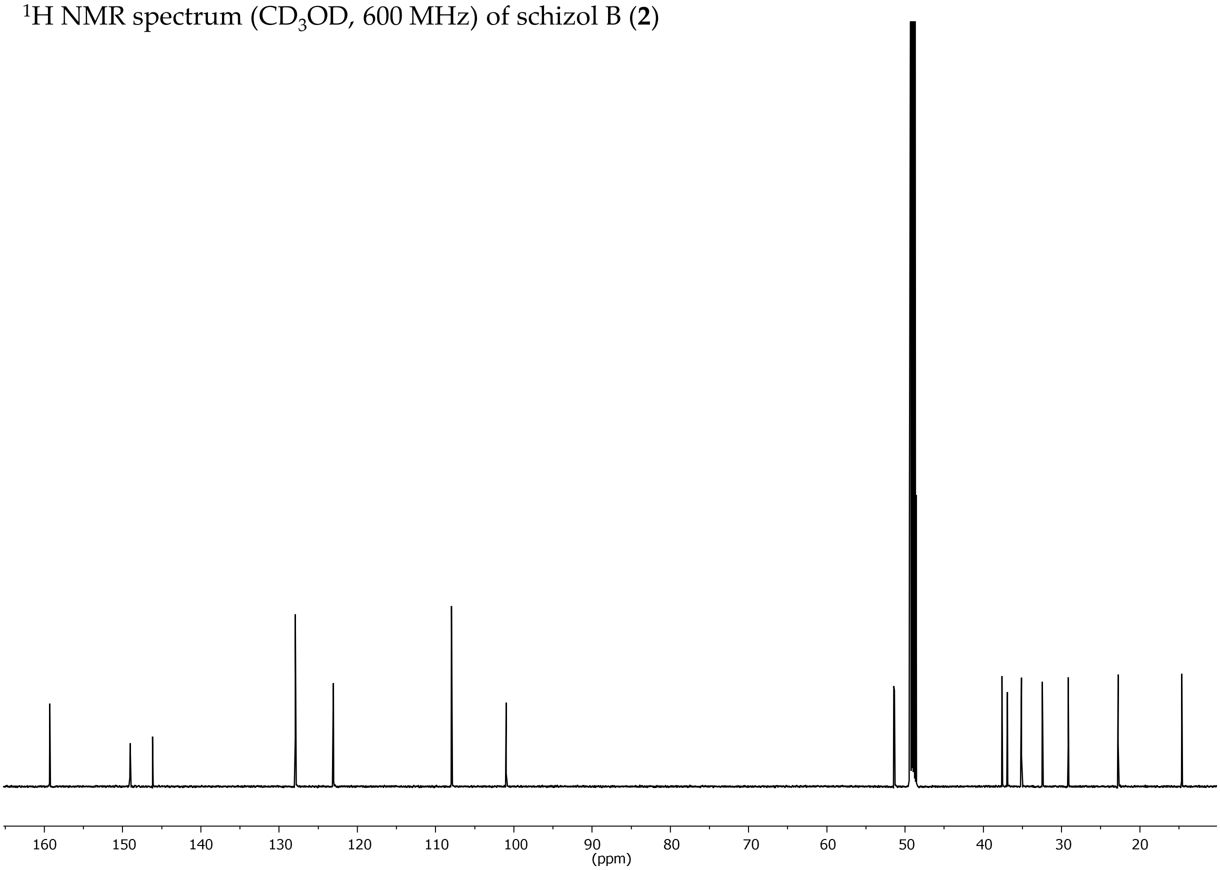

^{13}C NMR spectrum (CD_3OD, 150 MHz) of schizol B (**2**)

Figure S3

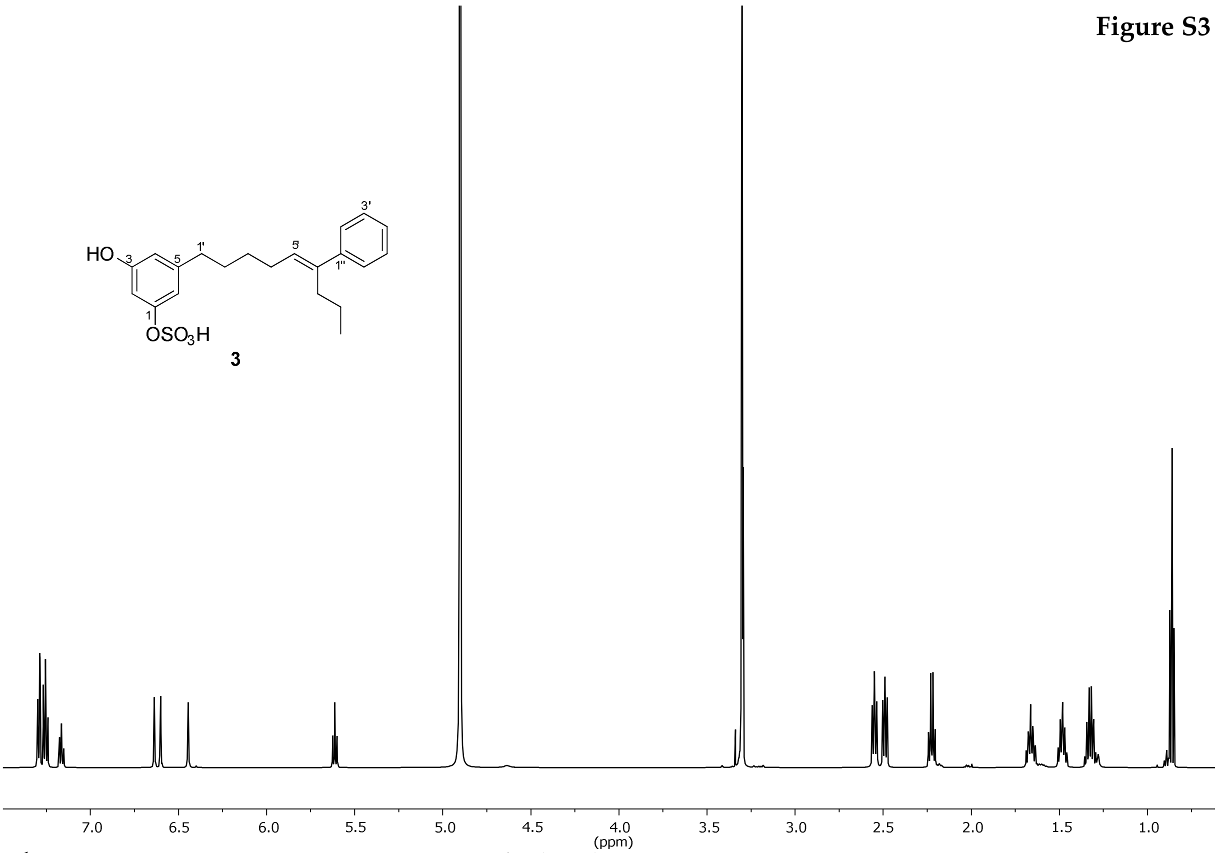

^{1}H NMR spectrum (CD_3OD, 600 MHz) of schizol C (**3**)

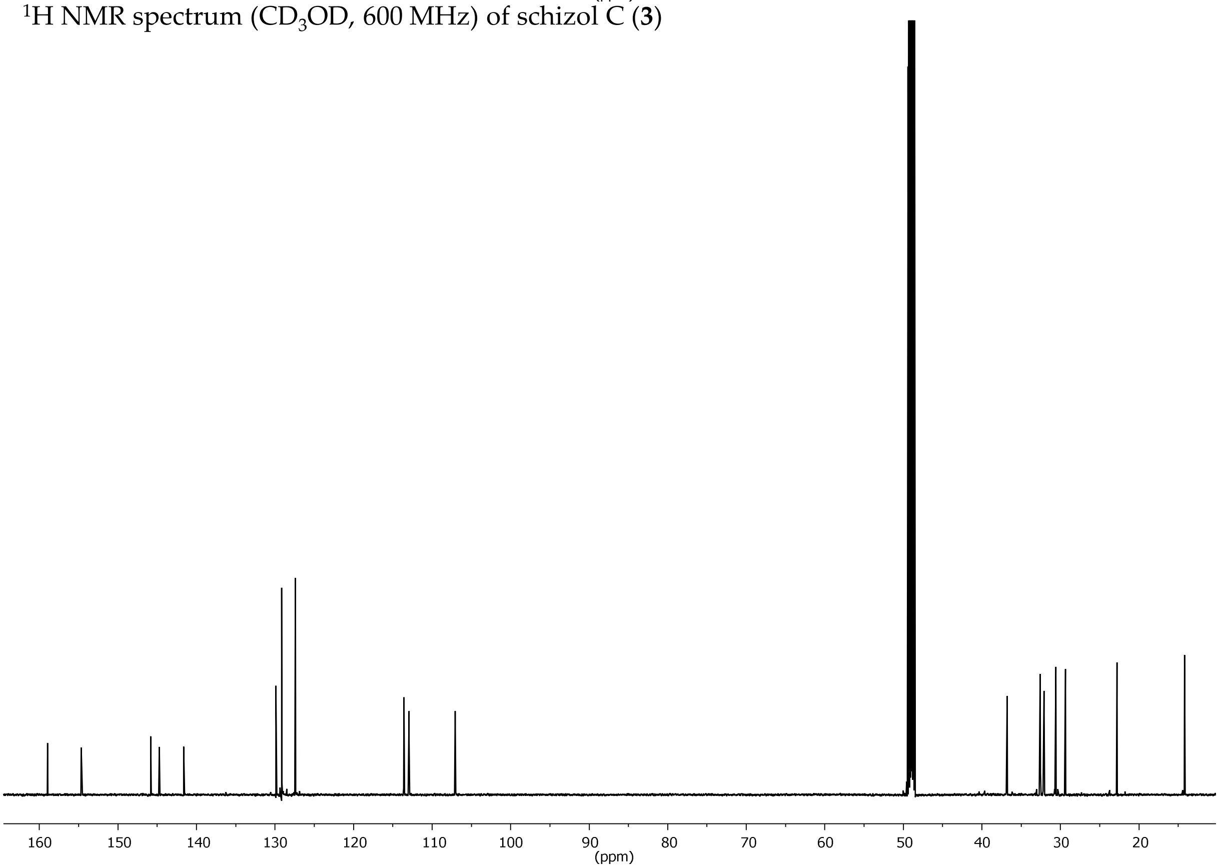

^{13}C NMR spectrum (CD_3OD, 150 MHz) of schizol C (**3**)

Figure S4

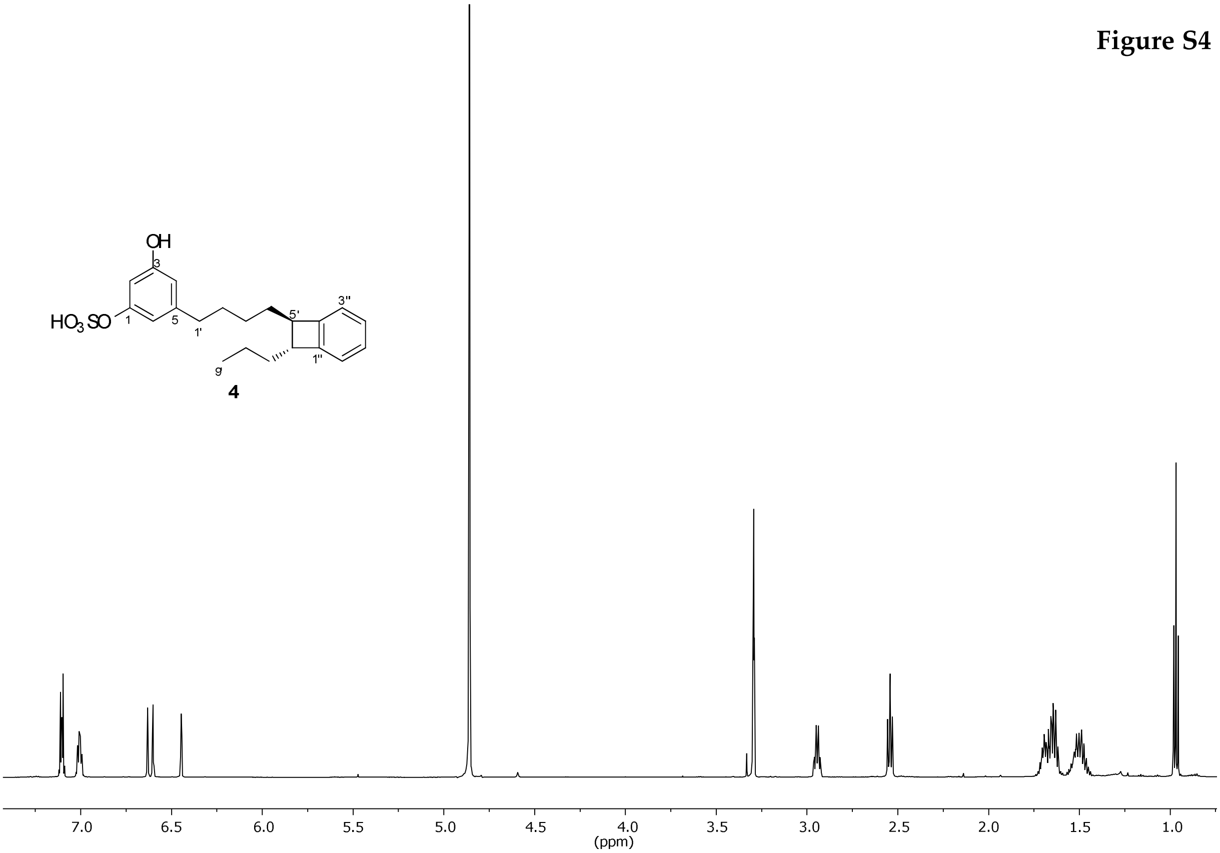

^{1}H NMR spectrum (CD_3OD, 600 MHz) of schizol D (**4**)

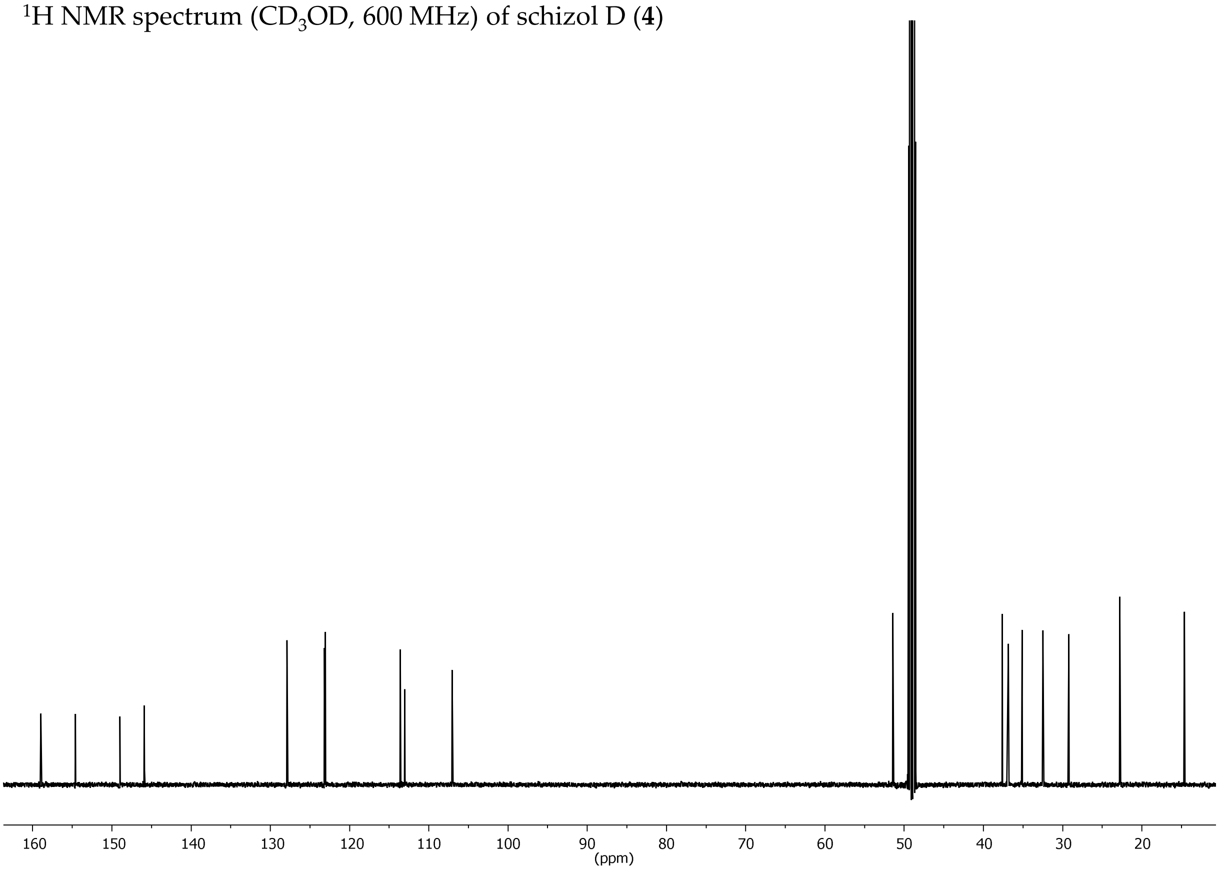

^{13}C NMR spectrum (CD_3OD, 150 MHz) of schizol D (**4**)

Figure S5

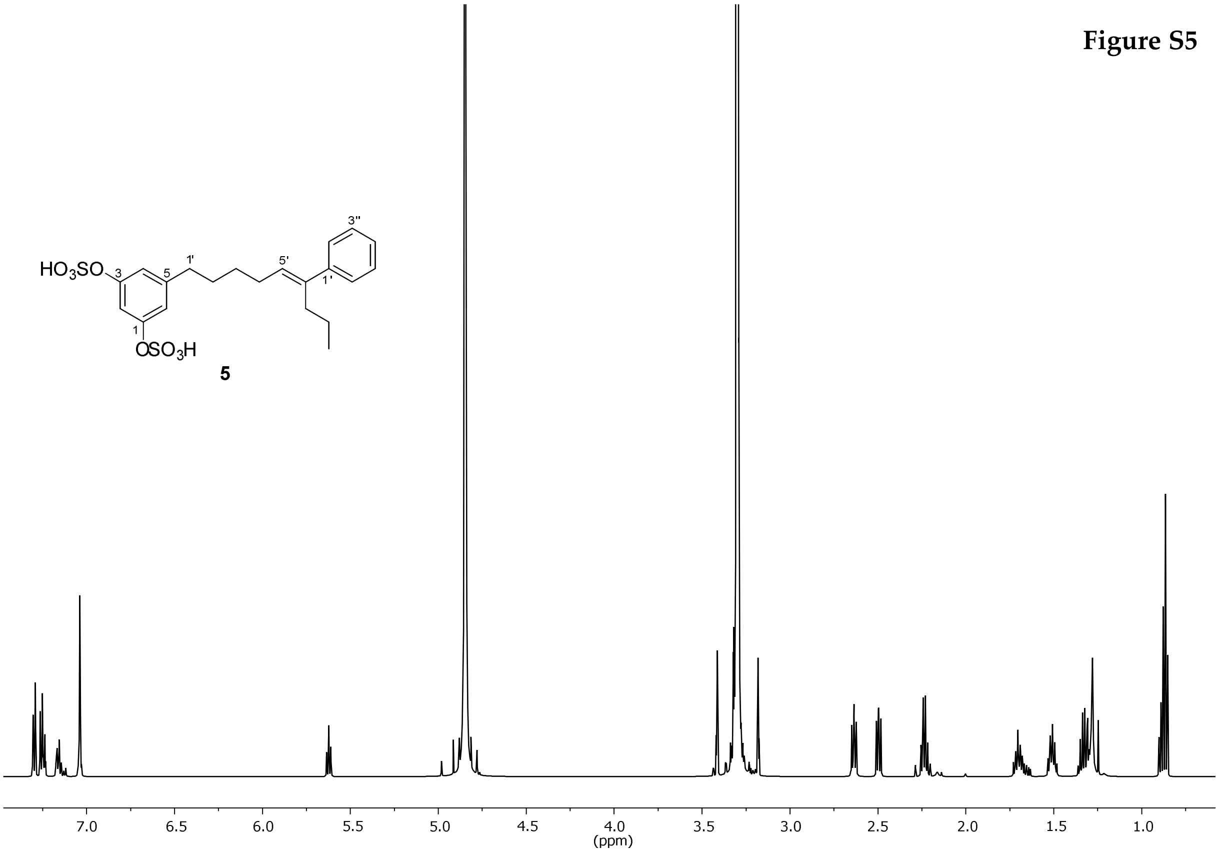

^{1}H NMR spectrum (CD_3OD, 600 MHz) of schizol E (**5**)

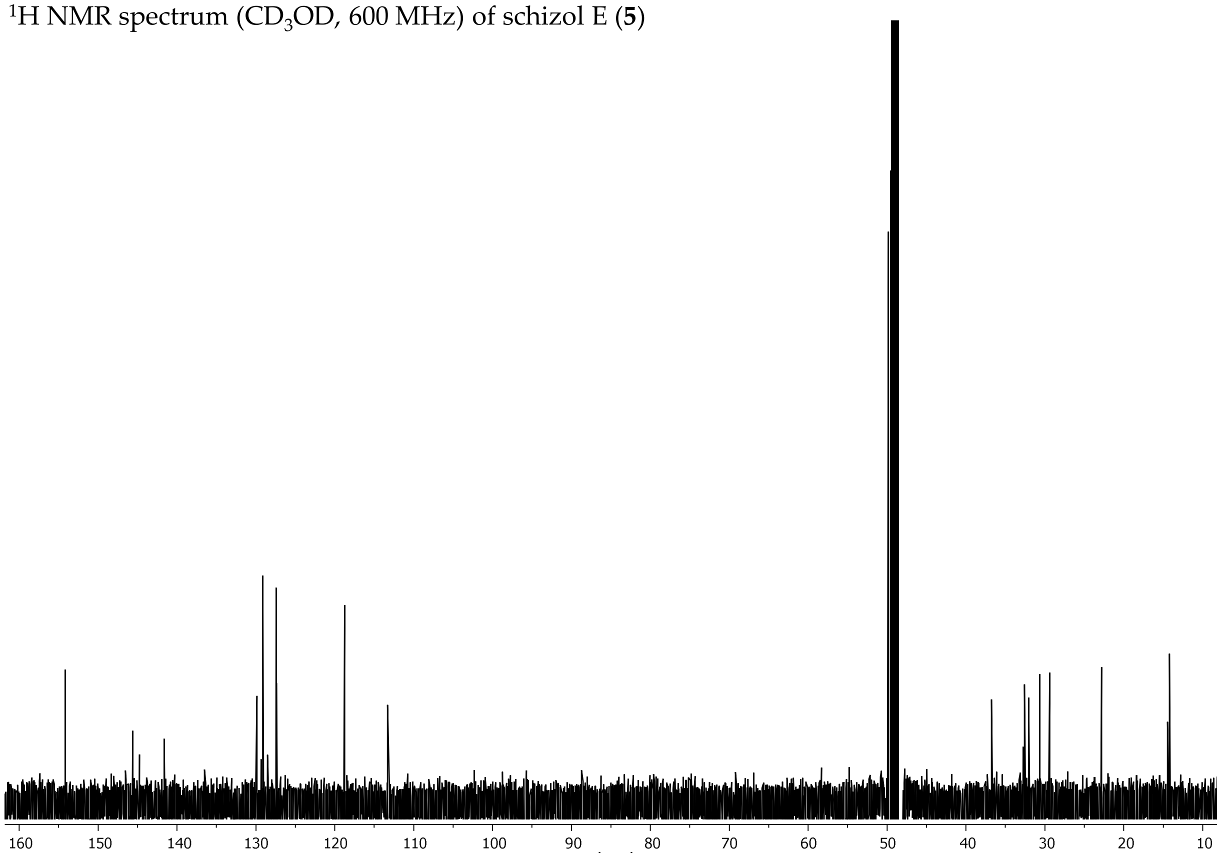

^{13}C NMR spectrum (CD_3OD, 150 MHz) of schizol E (**5**)

Figure S6

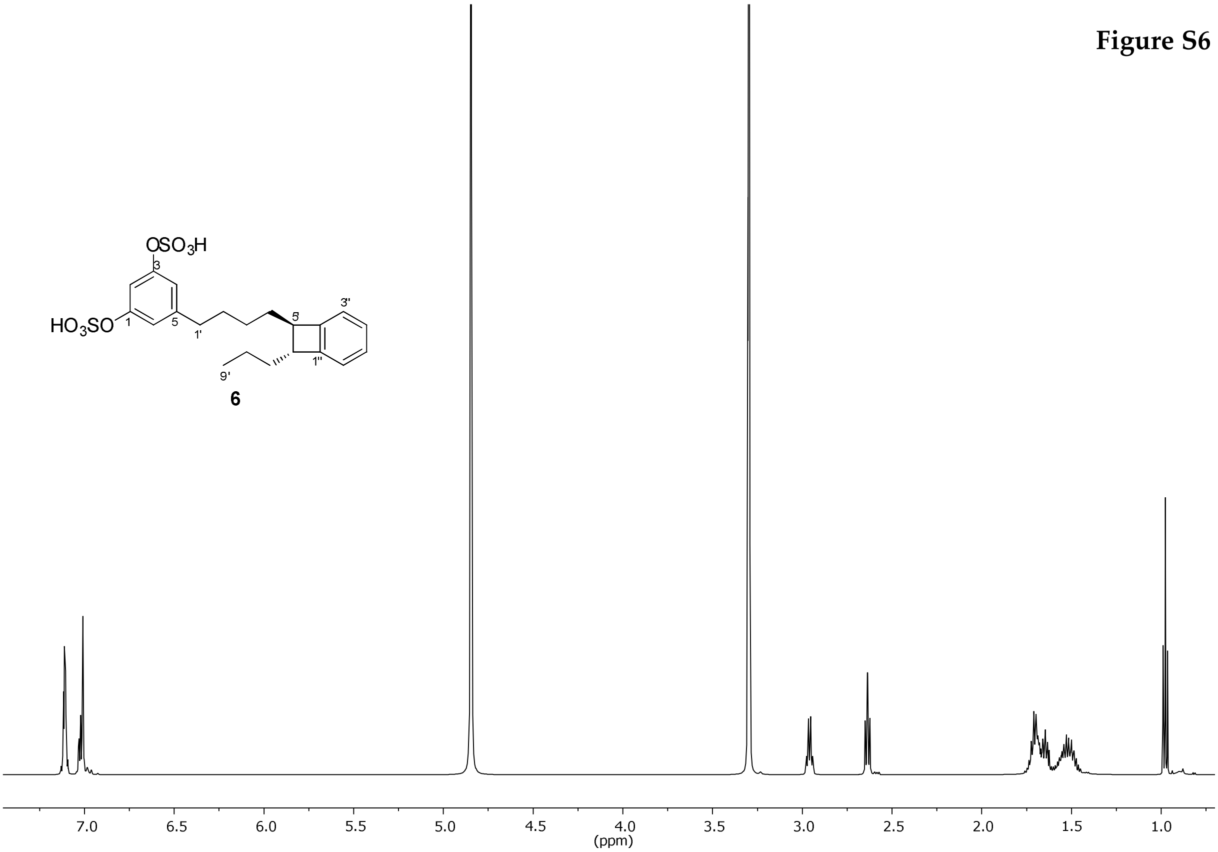

^{1}H NMR spectrum (CD_3OD, 600 MHz) of schizol F (**6**)

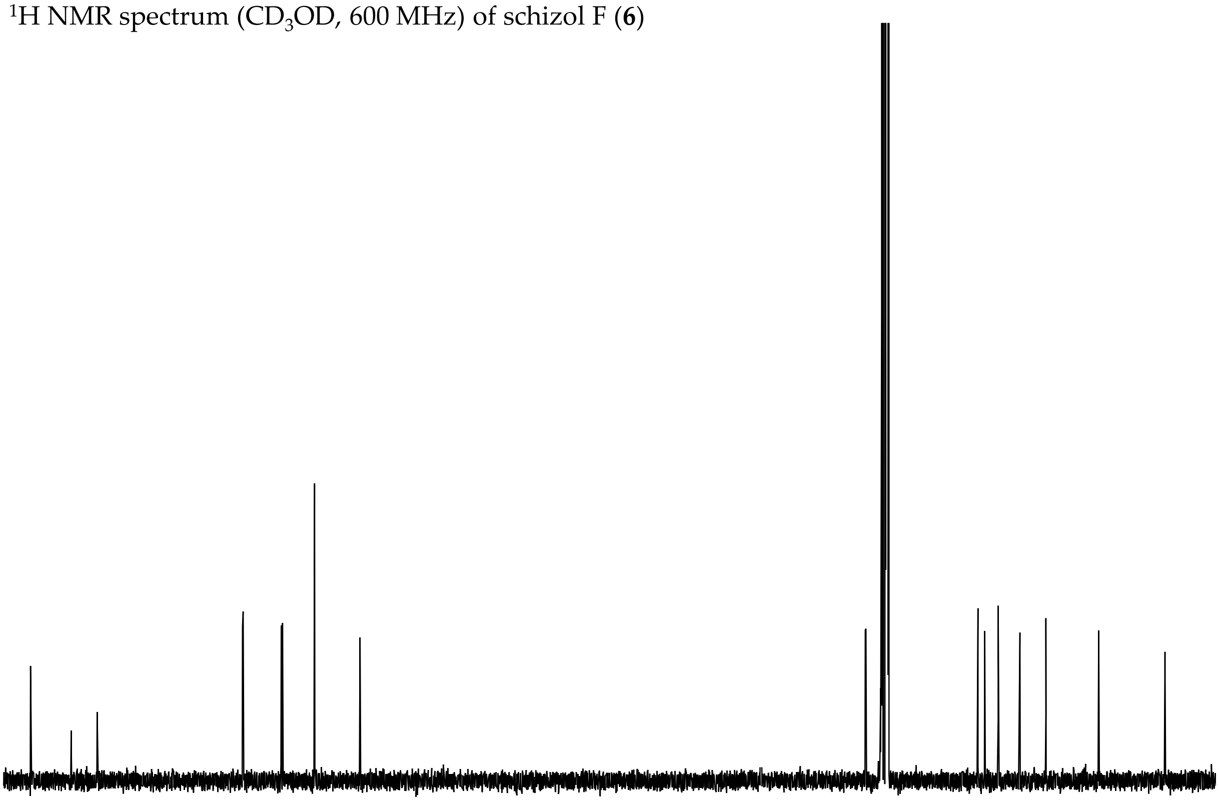

^{13}C NMR spectrum (CD_3OD, 150 MHz) of schizol F (**6**)

Synthesis of compound 7

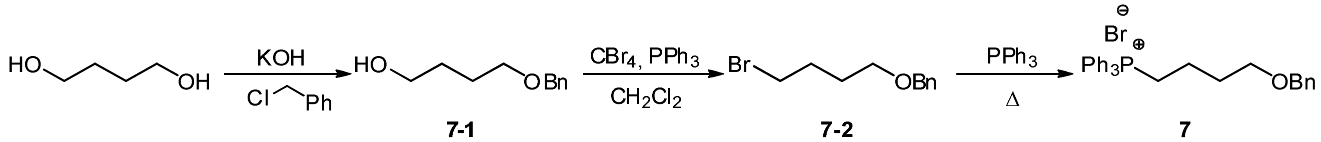

Compound **7-1**:

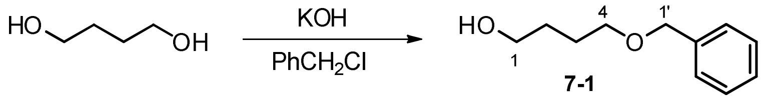

To 10 g of butane-1,4-diol (111.1 mmol) were added, at rt and under stirring, 4.48 g of KOH (80.0 mmol) and 5.1 mL of benzyl chloride (44.4 mmol) in 4 portions along 1h. After 4h, 30 mL of H_2O were added and the mixture was extracted with Et_2O (3 x 20 mL). The organic layers were combined, washed with H_2O (2 x 20 mL) and brine (20 mL), dried under anhydrous $MgSO_4$ and the solvent taken to dryness under reduced pressure, yielding 7.03 g of **7-1** (39.1 mmol, 88%) as a colorless oil. **^{1}H-NMR** (400 MHz, $CDCl_3$) δ 7.37-7.27 (m, 5H, H3′-H7′), 4.52 (s, 2H, H1′), 3.62 (t, *J* = 6.0 Hz, 2H, H1), 3.52 (t, *J* = 5.9 Hz, 2H, H4), 1.70 (m, 2H, H3), 1.67 (m, 2H, H2); **^{13}C-NMR** (100 MHz, $CDCl_3$) δ 128.3 (C4′ and C6′), 127.6 (C3′, C5′ and C7′), 138.3 (C2′), 72.9 (C1′), 70.2 (C4), 62.5 (C1), 29.9 (C2), 26.5 (C3); **IR** (film, cm^{-1}) 3354, 3030, 2939, 2866, 1495, 1453, 1362, 1092, 736, 697; **HRMS** (ESI) calcd for $C_{11}H_{16}O_2Na$: 203.1048 $[M+Na]^+$, found: 203.1060.

Compound **7-2**:

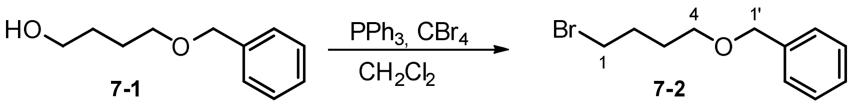

To a solution of 10 g of **7-1** (55.6 mmol) and 17.48 g of PPh_3 (66.7 mmol) in CH_2Cl_2 at 0 °C was added 20.28 g of CBr_4 (61.1 mmol). The resulting mixture was stirred at rt for 2h and then was concentrated under reduced pressure to give a residue that was purified by CC (hexanes/Et_2O 9:1) to yield compound **7-2** (10.93 g, 45.0 mmol, 81%) as a colorless oil. **^{1}H-NMR** (400 MHz, $CDCl_3$) δ 7.38-7.27 (m, 5H, H3′-H7′), 4.51 (s, 2H, H1′), 3.52 (t, *J* = 6.2 Hz, 2H, H4), 3.44 (t, *J* = 6.7 Hz, 2H, H1), 1.99 (m, 2H, H2), 1.78 (m, 2H, H3); **^{13}C-NMR** (100 MHz, $CDCl_3$) δ 128.3 (C4′ and C6′), 127.6 (C3′, C5′ and C7′), 138.4 (C2′), 72.9 (C1′), 69.2 (C4), 33.7 (C1), 29.7 (C2), 28.3 (C3); **IR** (film, cm^{-1}) 3030, 2860, 1495, 1453, 1363, 1104, 736, 697; **HRMS** (ESI) calcd for $C_{11}H_{15}O^{79}BrNa$: 265.0204 $[M+Na]^+$, found: 265.0205; calcd for $C_{11}H_{15}O^{81}BrNa$: 267.0184 $[M+Na]^+$, found: 267.0182.

Compound **7**:

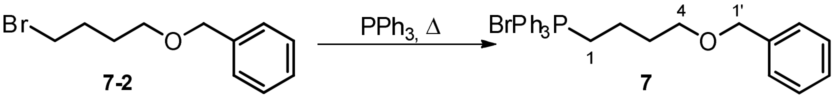

928 mg of **7-2** (3.82 mmol) and 1.0 g of PPh_3 (3.24 mmol) were heated overnight in an oven at 100 °C yielding 1.54 g of **7** (3.05 mmol, 80%) as an amorphous white solid. **^{1}H-NMR** (400 MHz, $CDCl_3$) δ 7.72-7.64 (m, 9H, -PPh_3), 7.60-7.54 (m, 6H, -PPh_3), 7.20-7.12 (m, 5H, H3′-H7′), 4.36 (s, 2H, H1′), 3.66 (m, 2H, H1), 3.50 (t, *J* = 5.7 Hz, 2H, H4), 1.89 (m, 2H, H3), 1.69 (m, 2H, H2); **^{13}C-NMR** (100 MHz, $CDCl_3$) δ 138.1 (C2′), 134.7, 133.3, 130.2 (PPh_3), 128.0 (C4′ and C6′), 127.4 (C3′, C5′ and C7′), 118.0 (PPh_3), 72.5 (C1′), 68.5 (C4), 29.3 (d, *J* = 16.2 Hz, C3), 21.6 (d, *J* = 50.3 Hz, C1), 19.3 (d, *J* = 3.9 Hz, C2); **IR** (film, cm^{-1}) 3055, 2866, 1587, 1438, 1113, 723, 691; **HRMS** (ESI) calcd for $C_{29}H_{30}OP$: 425.2034 $[M-Br]^+$, found: 425.2040.

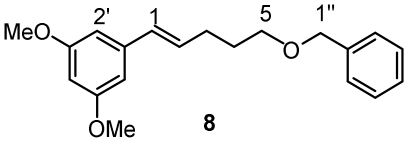

8

Compound **8 (isomer *E*):** **^{13}C-NMR** (100 MHz, $CDCl_3$) δ 160.9 (C3′ and C5′), 139.8 (C1′), 138.6 (C2′′), 130.8 (C2), 130.2 (C1), 128.3 (C4′′ and C6′′), 127.6 (C3′′ and C7′′), 127.5 (C5′′), 104.0 (C2′ and C6′), 98.8 (C4′), 72.9 (C1′′), 69.6 (C5), 55.3 (-OMe), 29.6 (C3), 29.3 (C4).

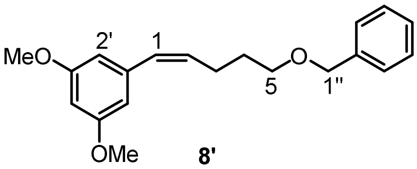

8'

Compound **8′ (isomer *Z*):** **^{13}C-NMR** (100 MHz, $CDCl_3$) δ 160.5 (C3′ and C5′), 139.4 (C1′), 138.5 (C2′′), 132.6 (C2), 129.3 (C1), 128.3 (C4′′ and C6′′), 127.5 (C3′′ and C7′′), 127.4 (C5′′), 106.8 (C2′ and C6′), 98.8 (C4′), 72.9 (C1′′), 69.7 (C5), 55.2 (-OMe), 29.9 (C4), 25.4 (C3).

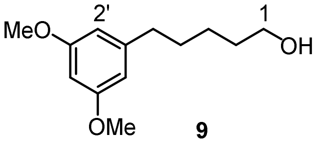

9

Compound **9: ^{13}C-NMR:** (100 MHz, $CDCl_3$) δ 160.6 (C3′ and C5′), 144.9 (C1′), 106.4 (C2′ and C6′), 97.5 (C4′), 62.7 (C1), 55.1 (-OMe), 36.1 (C5), 32.5 (C2), 32.0 (C4), 25.3 (C3).

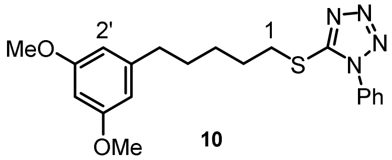

10

Compound **10: ^{13}C-NMR** (100 MHz, $CDCl_3$) δ 160.7 (C3′ and C5′), 130.0-123.8 (Ph), 154.4 (C1′′), 144.6 (C1′), 133.7 (Ph), 106.9 (C2′ and C6′), 97.7 (C4′), 55.2 (-OMe), 35.9 (C5), 33.2 (C1), 30.5 (C4), 29.0 (C2), 28.1 (C3).

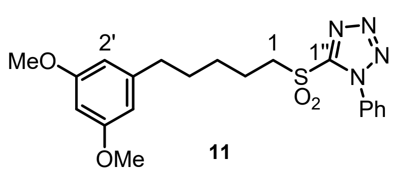

11

Compound **11: ^{13}C-NMR** (100 MHz, $CDCl_3$) δ 160.7 (C3′ and C5′), 131.4-125.0 (Ph), 153.4 (C1′′), 144.1 (C1′), 133.0 (Ph), 106.4 (C2′ and C6′), 97.8 (C4′), 55.9 (C1), 55.2 (OMe), 35.6 (C5), 30.4 (C4), 27.6 (C3), 21.9 (C2).

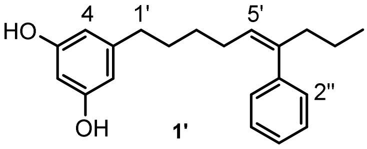

Compound **1′:** ^{13}**C-NMR** (150 MHz, $CDCl_3$) δ 159.3 (C1 and C3), 146.2 (C5), 142.6 (C1′′), 142.5 (C6′), 129.5 (C2′′ and C6′′), 129.1 (C3′′ and C5′′), 128.3 (C5′), 127.5 (C4′′), 107.9 (C4 and C6), 100.9 (C2), 42.5 (C7′), 36.7 (C1′), 31.8 (C2′), 30.7 (C3′), 29.6 (C4′), 22.2 (C8′), 13.9 (C9′).

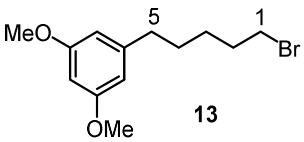

Compound **13**: ^{13}**C-NMR** (100 MHz, $CDCl_3$) δ 160.7 (C3′ and C5′), 144.6 (C1′), 106.4 (C2′ and C6′), 97.6 (C4′), 55.2 (-OMe), 35.9 (C5), 33.7 (C1), 32.6 (C2), 30.3 (C4), 27.7 (C3).

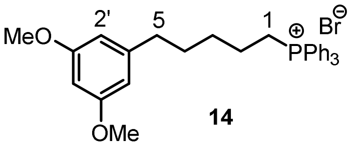

Compound **14**: ^{13}**C-NMR** (100 MHz, $CDCl_3$) δ 160.6 (C3′ and C5′), 144.5 (C1′), 134.9 (d, *J* = 3.2 Hz, C4′′), 133.5 (d, *J* = 12.3 Hz, C2′′ and C6′′), 130.4 (d, *J* = 12.3 Hz, C3′′ and C5′′), 118.2 (d, *J* = 85.42 Hz, C1′′), 106.3 (C2′ and C6′), 97.7 (C4′), 35.7 (C5), 30.6 (C4), 29.8 (d, *J* = 15.5 Hz, C3), 22.7 (d, *J* = 49.8 Hz, C1), 22.4 (d, *J* = 4.5 Hz, C2).

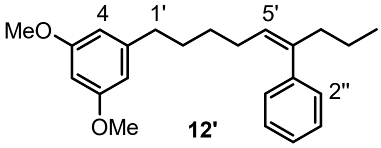

Compound **12′**: ^{13}**C-NMR** (100 MHz, $CDCl_3$) δ 160.6 (C1 and C3), 145.2 (C5), 141.5 (C1′′), 140.9 (C6′), 128.4 (C2′′ and C6′′), 127.9 (C3′′ and C5′′), 127.1 (C5′), 126.2 (C4′′), 106.4 (C4 and C6), 97.6 (C2), 41.4 (C7′), 36.0 (C1′), 30.6 (C2′), 29.7 (C3′), 28.6 (C4′), 21.2 (C8′), 13.6 (C9′).
